# Genetic diversity and adaptability of native sheep breeds from different climatic zones

**DOI:** 10.1038/s41598-025-97931-2

**Published:** 2025-04-23

**Authors:** George Wanjala, Zoltán Bagi, Dinu Gavojdian, Bouabid Badaoui, Putri Kusuma Astuti, Alexandru Mizeranschi, Elena Ilisiu, Husein Ohran, Eva Pasic Juhas, Dimitrios Loukovitis, Aldona Kawęcka, Rūta Šveistienė, Zsolt Becskei, Péter Strausz, Nelly Kichamu, Szilvia Kusza

**Affiliations:** 1 Centre for Agricultural Genomics and Biotechnology, Faculty of Agricultural and Food Sciences and Environmental Management, University of Debrecen, Egyetem tér 1, Debrecen, 4032 Hungary; 2 Doctoral School of Animal Science, University of Debrecen, Böszörményi út 138, Debrecen, 4032 Hungary; 3 Institute of Animal Sciences and Wildlife Management, University of Szeged, Andrássy út 15, Hódmezővásárhely, 6800 Hungary; 4 Research and Development Institute for Bovine, Romanian Academy of Agricultural and Forestry Sciences, sos Bucuresti-Ploiesti km 21, Balotesti, 077015 Romania; 5 Laboratory of Biodiversity, Ecology, and Genome, Department of Biology, Faculty of Sciences, Mohammed V University in Rabat, Rabat, Morocco; 6 African Sustainable Agriculture Research Institute (ASARI), Mohammed VI Polytechnic University (UM6P), Laâyoune, Morocco; 7 Research and Development Station for Bovine, Bodrogului 32, Arad, 310059 Romania; 8 Institute for Advanced Environmental Research, West University of Timişoara, Str. Oituz nr. 4, Timişoara, Romania; 9 Research and Development Institute for Sheep and Goat Palas - Constanta, I.C. Brătianu, 248, Constanţa, Romania; 10 Department of Physiology, University of Sarajevo – Veterinary Faculty, Zmaja od Bosne 90, Sarajevo, 71000 Bosnia and Herzegovina; 11 Department of Fisheries and Aquaculture, School of Agricultural Sciences, University of Patras, New buildings, Mesolongi, 30200 Greece; 12 Department of Sheep and Goat Breeding, National Research Institute of Animal Production, Balice n., Cracow, 32-083 Poland; 13 Animal Science Institute, Lithuanian University of Health Sciences, Baisogala, 82317 Lithuania; 14 Department of Animal Breeding and Genetics, Faculty of Veterinary Medicine, University of Belgrade, Bulevar Oslobodjenja 18, Belgrade, 11000 Serbia; 15 Institute of Strategy and Management, Department of Management, Corvinus University of Budapest, Fővám tér 8, Budapest, 1093 Hungary; 16 Ministry of Agriculture Livestock, Fisheries, and Cooperatives, State Department of Livestock Development, Naivasha sheep and goats breeding station, Box 20117, Naivasha, Kenya

**Keywords:** Genetics, Agricultural genetics, Animal breeding, Genetic markers, Genomics

## Abstract

To better understand the genetic architecture and adaptability of native sheep, 22 breeds were genotyped using ovine50K SNP chips. Eleven additional populations from open-source repositories were included. Cross population Extended Haplotype Homozygosity (XP-EHH) and Relative Scaled Haplotype Homozygosity (Rsb) haplotype-based approaches were used to identify genetic variations influencing the adaptation of local sheep breeds to different climatic zones. The results indicate that all breeds exhibited high but declining genetic diversity levels, with a larger proportion of genetic variation explained by development history and refined by geographical origin, as shown by principal component analysis and Neighbor Net graphs. Admixture analysis revealed high admixture levels in European and North African breeds. Using XP-EHH and Rsb methods, 371 genes were putatively under selection, with only nine common among all population pairs, highlighting unique adaptability. Most identified genes, including Interleukin (IL) and cluster differentiation (CD) gene families underlie immune responses, emphasizing their role in resilience to climate change effects. These findings support that indigenous sheep breeds have retained significant genetic diversity, but improper genetic management may threaten it. Additionally, the research emphasizes that indigenous sheep breeds’ unique adaptability to specific climatic zones makes them valuable genetic assets for developing climate-resilient breeds.

**Supplementary Information:**

The online version contains supplementary material available at 10.1038/s41598-025-97931-2.

## Introduction

Sheep are one of the domestic livestock species that significantly contribute to both the world’s food security and animal-derived proteins. They were domesticated in the Fertile Crescent of western Asia more than 11,000 years ago^1^. Sheep have a broad geographic distribution, inhabiting several ecological zones all around the globe. This species displays a range of morphological characteristics that are hypothesized to have arisen as adaptations to the specific ecological conditions of their respective habitats^2^. Beyond their primary products of meat, milk, and wool^3^, sheep offer additional products such as manure and the ability to utilize marginal lands. In many developing countries, especially within Africa, rural communities rely heavily on sheep for their primary animal protein source and as a measure of wealth. These communities often favor native breeds due to their resilience and adaptability to local breeding conditions^4^. In contrast to other major livestock species like poultry and pigs, which are predominantly raised in intensive conditions, native sheep breeds are primarily managed under extensive systems, making them more susceptible to the impacts of climate change. Given that most native sheep breeds are frequently overlooked in commercial contexts and that their breeding practices are not systematically managed, the absence of structured breeding programs results in frequent random mating. This, in turn, can lead to elevated levels of inbreeding and a subsequent decline in genetic diversity. Nevertheless, scholars contend that, due to their limited exposure to artificial selection and crossbreeding, native sheep breeds maintain a rich genetic diversity^5^.

Over 1300 indigenous sheep breeds have been identified globally, with a significant proportion distributed across Africa, Asia, and Europe^6,7^. In contrast, prominent sheep breeding regions like Australia and New Zealand predominantly focus on a selected few elite breeds, such as the Merino and Romney. These native breeds not only exhibit vast genetic diversity but also possess potential genomic regions that underpin their adaptability to various climatic challenges. These challenges encompass heat stress tolerance, resistance to diseases, and adaptability to arid feeding conditions^5,8,9^.

Understanding the genomic mechanisms that facilitate adaptation is crucial for the effective management of sheep genetic resources. Some hypotheses suggest that geographical isolation and adaptability to diverse climatic zones differentiate native sheep breeds, aligning them with the climatic zones of their origin. This perspective contrasts with Kijas^10^ study, which found minimal phylogeographical clustering among global sheep breeds. It is postulated that Kijas inclusion of a substantial number of mainstream and transboundary sheep breeds might have influenced breed clustering, leading to potential biases in the interpretation of results.

Given the pronounced phenotypic variance observed in native sheep breeds and their significant exposure to natural selection, we hypothesize that breed divergence and evolutionary adaptation in native breeds have been primarily driven by ecological factors. Despite the importance of understanding the genomic evolution of native sheep breeds in relation to different climatic zones, comprehensive studies in this domain remain limited. While some recent research endeavors have sought to elucidate this concept^2,11–14^, many were either region-specific or incorporated non-native breeds in their analyses.

In light of these gaps, this study, utilizing 50 K ovine SNP data, aims to:

1. Ascertain the genetic diversity within and between native sheep breeds from distinct climatic regions.
2. Determine the genetic relationships among native sheep breeds.
3. Identify signatures indicative of adaptation to contrasting climatic zones.

Objectives 1 and 2 specifically targeted recently genotyped indigenous sheep breeds, which were genotyped specifically for the purpose of this study. Objective 3, on the other hand, encompassed other breeds that were collected from public sources.

## Results

### Genetic diversity within and between breeds

Upon examining the genetic diversity both within and amongst various sheep breeds, it becomes evident that the standard diversity indices present a consistent pattern. Specifically, the *Ho* and *He* levels across the studied breeds were strikingly similar. The *Ho* values for the 33 sheep breeds under investigation ranged from 0.284 (SWE.Fja) to 0.393 (HUN.Tetra). Concurrently, the *He* values oscillated between 0.331 (SWE.Dal) and 0.396 (SWE.Fja). Furthermore, there were discernible levels of inbreeding, as evidenced by the F coefficients, which varied between 0.025 (ROU.Rtsig) and 0.514 (SWE.Fja; see Supplementary File 1 Table S1).

The values of recent effective population size for at most 45 generations ago (N_13_ and N_45_) unveiled a pronounced decline in the effective population. Values of Ne_13_ ranged between 32 (SWE.Klo) and 224 (KEN.Eaft) while Ne_45_ ranged between 88 (SWE.Klo and 650 (ETH.Menz). The highest decline between N45 and N13 was observed in KEN.Eaft (465) while the lowest was observed in LTU.Cwool (43).

It’s noteworthy that a significant number of European breeds exhibited an Ne_13_ of less than 100. However, there were exceptions, namely BIH.DPram, GRC.Bout, HUN.IldeFr, HUN.Mer, ROU.Turc and ROU.Rtsig, all of which demonstrated a *Ne* exceeding 100. In contrast, all of African and Asian breeds boasted an *Ne* greater than 100. Intriguingly, the SWE.Klo and SWE.Dal, LTU.Cwool, and HUN.Dor all exhibited an Ne_13_ of below 50.

### Population structure, relationship and breed divergence

**Fig. 1:**
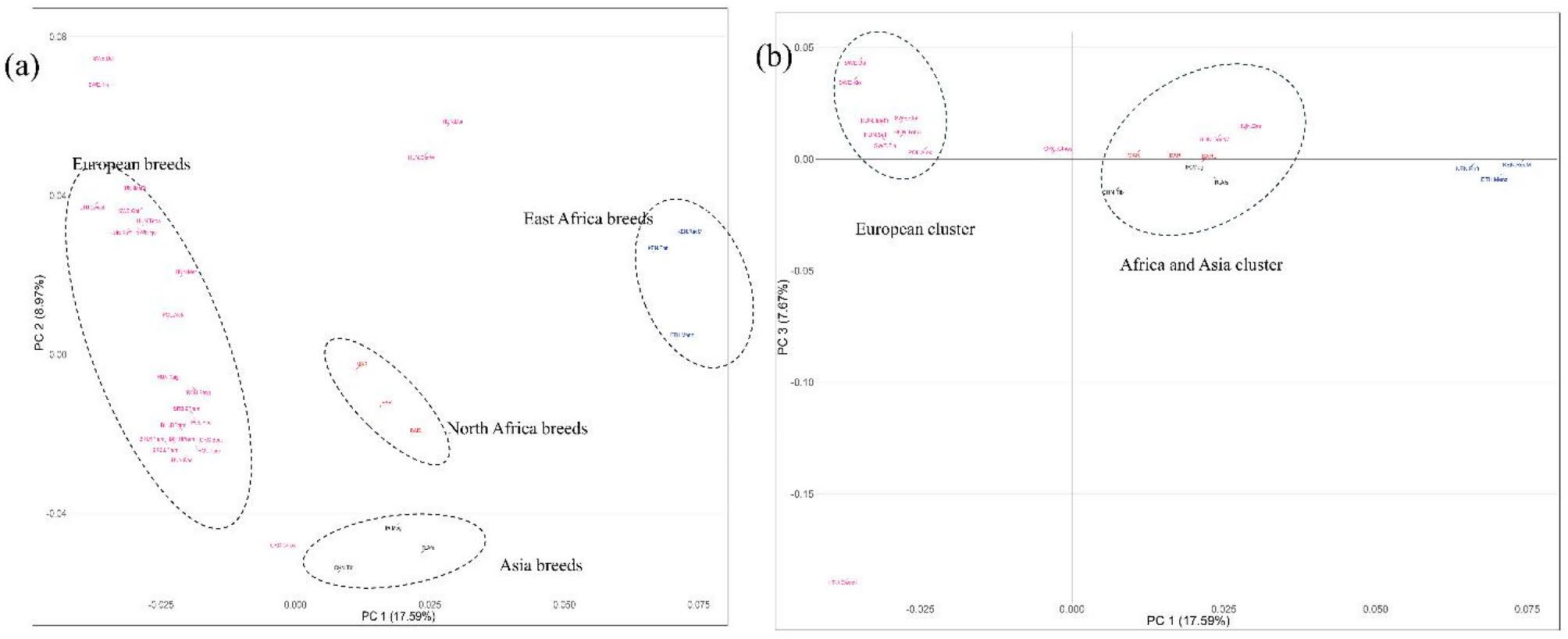
Principal Component Analysis for all the 33 studied sheep breeds. (**a**) Principal Components 1 and 2; (**b**) Principal Components 1 and 3.

The determine the source of genetic variation in these breeds, we performed ANOVA analysis. The results derived from the AMOVA analysis elucidated that a substantial 91% of the genetic variation emanated from the variations among individuals within the breed. Conversely, a mere 8% (*p* < 0.001) of this diversity is attributed to among-breed genetic variations (see Supplementary File 1 Table S2). Further, we also investigated the genetic relationship between breeds using principal component analysis. Figure 1 presents the genetic positions of and relationships among breeds under principal components (PCs). The first three PCs accounted for only 34% of the genetic diversity between all breeds. Principal component one accounted for 17.59, PC 2 accounted for 8.97 while PC3 accounted for 7.67 of the genetic diversity. The three PCs cluster breeds based on their geographical proximity (in this case, refined by climatic regions) or genetic history. PC1 and PC2 (Fig. 1a) clearly differentiated breeds from tropical regions (East Africa; KEN.RedM, KEN.Eaft, and ETH.Menz), North African breeds (North Africa; MAR and BAR and BARL), Asian breeds (CHN.Tib, IR.Mog, and IR.Afs), and most European breeds displayed less distinct clustering patterns. HUN.Dor.W and HUN.Dor are aligned with East African breeds, but GRC.Chios stands out as being genetically correlated with Asian breeds (Fig. 1a). Surprisingly, LTU.Cwool forms a separate cluster along PC 3 (Fig. 1b), while two distinct clusters are created, one consisting of European breeds and the other consisting of African and Asian breeds.

**Fig. 2:**
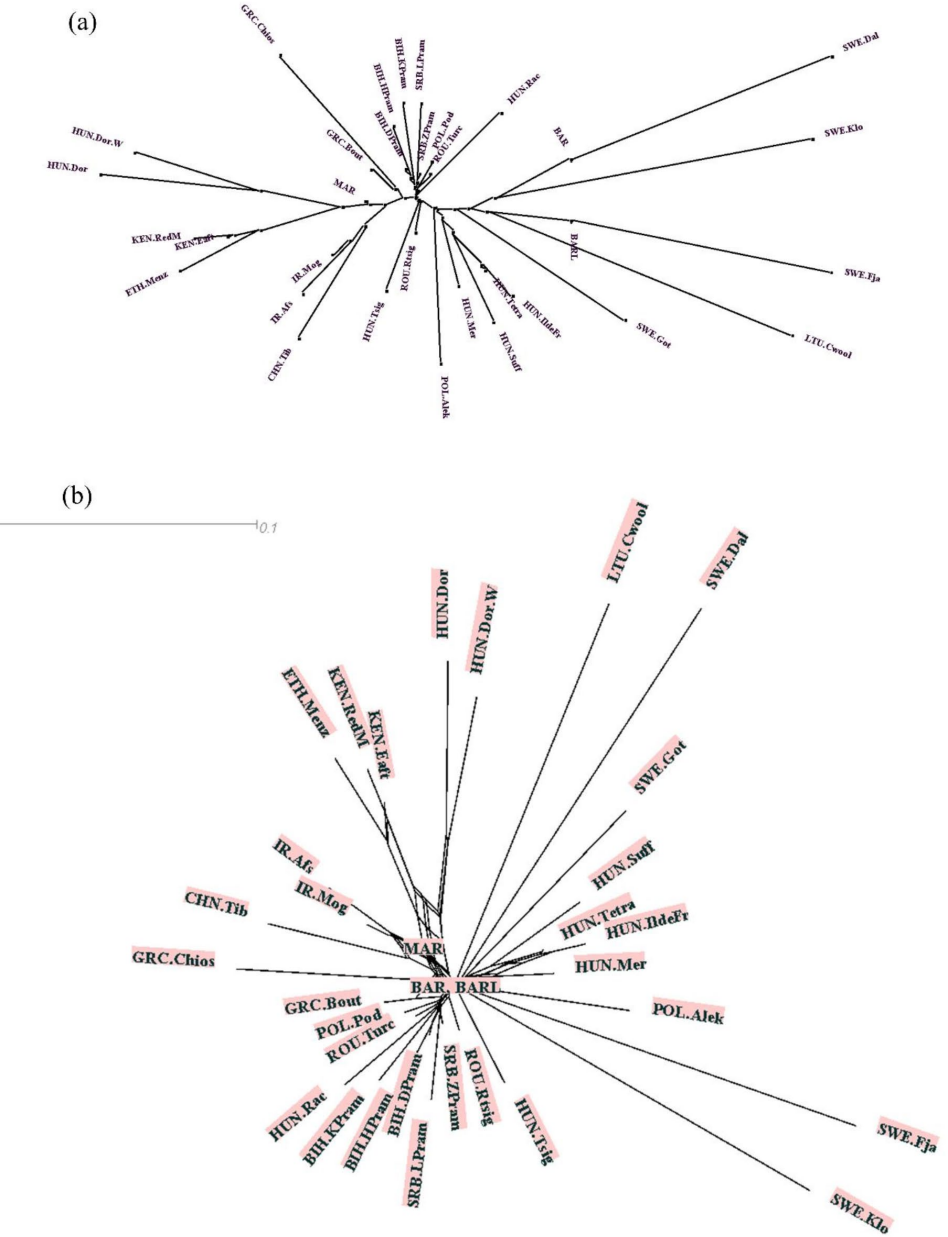
Relationship between native sheep breeds. (**a**) based on Reynolds distance; (**b**) based on divergence time.

Relationship between breeds were examined using Fst matrix, relationship between breeds based on Reynold’s genetic distance and divergence time. Fst values of all pairs of breeds ranged between 0.006 (BAR vs. BARL-least differentiated breeds from each other) and 0.304 (SWE.Fja and SWE.Dal- most differentiated from each other). All Fst scores differed significantly (*p* < 0.05) from each other. NeibourNet graphs were constructed based on Reynold’s genetic distances (Fig. 2a) and divergent time (Fig. 2b) using split tree software^13^.

Figure 2a reveals the presence of three primary clusters, each including several subclusters. These clusters are primarily determined by both geographical origin and historical history. Geographically, breeds coming from Asia and Africa, such as HUN.Dor and HUN.Dor.W, but excluding BAR and BARL, are clustered together on one branch. These breeds also have a close connection to breeds originating from Greece. The remaining two clusters consist of European nations’ breeds, but cluster membership is possibly refined by their developmental histories. Interestingly, BAR and BARL are clustered close to Sweden breeds. Similar clusters are observed in Fig. 2b except North African breeds are positioned at the center of the NeibourNet, suggesting that they have a close relationship with many populations under investigation. On the other hand, SWE.Fja, SWE.Klo, SWE.Dal, SWE.Got, LTU.Cwool appear to be highly genetically differentiated compared to other populations.

**Fig. 3:**
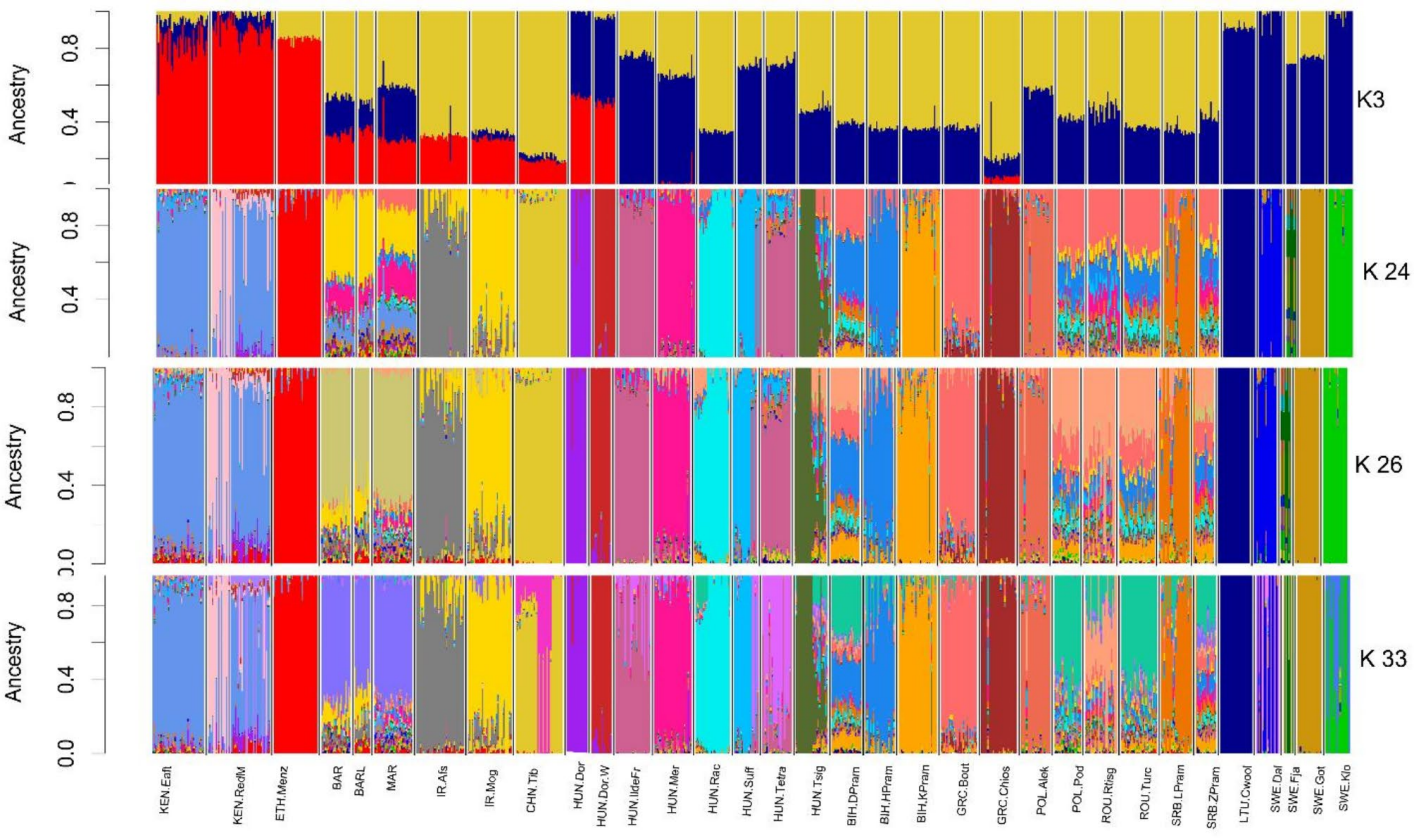
Genome-wide histogram of ancestry analysis for K = 3, 24, 26 and 33.

To determine the proportion of shared ancestral components, admixture analysis was performed. The cross-validation entropy (CV) of the ancestry proportion analysis suggested that the optimal cluster number for the ancestry analysis of the 33 breeds, using the admixture software, stands at 26 (see Supplementary File 2, Figure S1a). The admixture pattern obtained at K = 3 (representing 3 continents- Asia, Africa and Europe), K = 24 (CV at which the curve started to flatten), K = 26 (The lowest point of the CV curve and K = 33 (Number of separate breeds involved in the study) is presented in Fig. 3. At K = 3, except for the East African and Swedish breeds which showed somehow homogenous populations, all other breeds shared ancestral blood between Asian and European breeds. At K = 26, some breeds like LTU.Cwool, SWE.Got, SWE.Klo, HUN.IldeFr, HUN.Dor, HUN.Dor.W, CHN.Tib and ETH.Menz exhibited homogenous groups, and the rest showed elevated levels of admixture. The highly admixed group of breeds was North African breeds which shared genetic components with Kenyan, Iranian, GRC.Bout and HUN.Dor.W. Other breeds like ROU.Rsig, ROU.Turc, BIH. Dpram and SRB.Zpram exhibited similar admixture patterns, sharing ancestral components with multiple breeds. A similar pattern is also observed at K = 33. See Supplementary File 2: Figure S1b for More detailed presentation of shared ancestral proportion between K = 2 and K = 35.

### Selection signatures for local environmental adaptation

The identification of signatures of selection for local adaptation was performed utilizing two haplotype approaches, namely Rsb and XP-EHH. Genes that were found by both approaches in each pair of population groups were chosen. To summarize, the comparison between North and East African breeds resulted in the identification of 80 common genes (Rsb = 112, XP-EHH = 125). Comparing East African breeds with European breeds led to the discovery of 202 common genes (Rsb = 254, XP-EHH = 277), while comparing North African breeds with European breeds revealed 89 common genes (Rsb = 132, XP-EHH = 112).

**Fig. 4:**
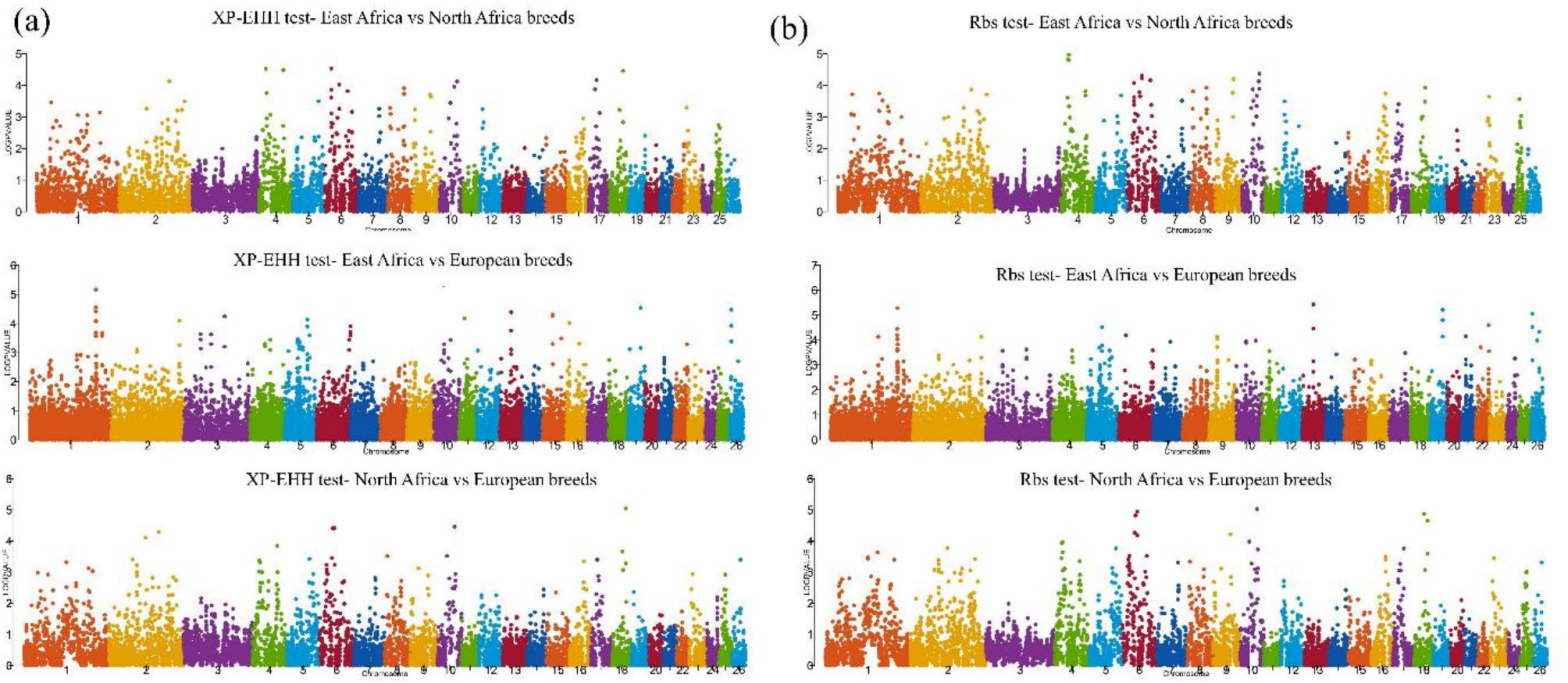
Manhattan plots of the (**a**) XP-EHH and (**b**) Rbs tests in the comparison between populations from contrasting climatic zones (labeled).

Genomic hotspots were detected on multiple chromosomes in all population pairings, indicating that adaptability is a multifaceted trait that depends on numerous genes (Fig. 4).

Gene-gene interaction network analysis and biological process and pathway enrichment analysis were conducted on the common genes identified in each population pair. The GeneMANIA (https://genemania.org^15^) was employed to analyze the network of interactions between genes, specifically gene-gene interactions. On the other hand, the Metascape^16^, also used by Qiao et al.^16^ was utilized for gene ontology and enrichment terms. Results for gene-gene interaction network obtained from GeneMania and Metascape are shown in Supplementary File 3;Figures S2–. The comparison of gene expression and physical interaction between East African and European native sheep populations revealed a co-expression rate of 41.86% and a physical interaction rate of 33.80%. In the case of North African vs. European comparison, the physical interaction rate was 41.66% and the co-expression rate was 26.90%. Lastly, when comparing East African vs. North African populations, the co-expression rate was 41.90% and the physical interaction rate was 19.99%. The heatmap analysis revealed that the enrichment of common genes varied among different population pairs. Notably, the most significant gene ontology (GO) terms in East Africa vs. European populations were related to cytokine signaling in the immune system and the control of immune tolerance by vasoactive intestinal peptide. In contrast, both the East Africa vs. North Africa and North Africa vs. European population pairs showed the most significant GO term to be positive regulation of protein modification process.

## Discussion

Indigenous sheep breeds predominantly thrive under extensive natural production systems, frequently confronting a myriad of climatic and environmental adversities. These challenges encompass heightened parasitic loads, significant pathogenic pressures, suboptimal feed quality, periodic feed shortages, and temperature extremes. Additionally, these breeds often suffer from subpar breeding methodologies. Consequently, delineating the genetic architecture of these indigenous breeds and deciphering their inherent genetic mechanisms of adaptability becomes imperative. Such insights pave the way for advanced molecular-driven genetic enhancement initiatives and climate-resilient selection strategies. Through comprehensive genome-wide analyses, scholars have the potential to unravel genetic diversity, discern population dynamics, and identify novel genomic regions and mutations that facilitate adaptation to local climate. For such endeavors to be successful, it is essential that SNP markers comprehensively span the genome and exhibit polymorphism within each population. In this study, 50 K ovine autosomal SNPs data was analyzed, which, based on our empirical evidence, emerged as a robust molecular tool for a thorough population genomics exploration in sheep breeds.

### Intra-population genetic diversity

In our comprehensive analysis, all the native breeds and outgroups under investigation demonstrated relatively consistent and high levels of heterozygosity (see Supplementary File 1: Table S1). The *Ho* ranged from 0.284 to 0.393, while the *He* spanned from 0.331 to 0.396. These findings agree with the results from the Merino group, encompassing both Merino and Merino-derived breeds, as explored by Ciani et al.^17^ and subsequently re-evaluated by Zsolnai et al.^18^. Similar patterns were also evident in global goat breeds as documented by Colli et al.^19^.

While we initially hypothesized that breeds such as IR.Afs and IR.Mog would exhibit higher heterozygosity levels, given their decreased distance from the domestication center, certain European native breeds surprisingly manifested higher heterozygosity than these breeds. The F_HOM_ derived from *Ho* and *He* exceeded the expected 0.055 for the majority of the sheep breeds under study. However, seven specific breeds, namely, BAR, BARL, BIH.DPram, HUN.Tetra, POL.Pod, ROU.Rtsig, ROU.Turc, and SRB.Zpram, recorded F_HOM_ values less than 0.055. Therefore, except for the later seven breeds, all other breeds have experienced higher levels of inbreeding than expected. Such elevated inbreeding levels were expected, given the prevalent mob breeding practice among local sheep, where indigenous sheep are bred *en masse* without observing selection. Persistently high inbreeding can be detrimental in terms of evolution, potentially leading to the adverse consequences associated with inbreeding depression^20^. Historically, inbreeding within populations was managed using pedigree data^21^. However, given the infrequent record-keeping in native sheep breeds, molecular estimation of inbreeding levels emerges as a promising avenue to enhance breed management and mitigate inbreeding risks.

All breeds exhibited a rapid decline of *Ne* between Ne_45_ and Ne_13_ generations. In other words, the *Ne* values for 45 generations were higher than that of 13 generations (see Supplementary File 1: Fig. S 1). Notably, the most recent *Ne* of some European sheep populations was below 100 about 13 generations ago except for ROU.Turc, ROU.Tsig, GRC.Bout, and BIH.Dpram. The present results agree with the research conducted by Nosrati et al.^22^. In contrast, all breeds originating from Africa and Asia exhibited Ne_13_ exceeding 100. Drawing from Taberlet et al.^23^ the International Union for Conservation of Nature (IUCN) classifies species or breeds based on their extinction vulnerability. Populations deemed critically endangered have a *Ne* below 50, those categorized as endangered have an *Ne* below 250, and those labeled vulnerable exhibit an *Ne* below 1000. Lynch & Lande^24^ advocate for most recent *Ne* to be 500 to stave off long-term population extinction. Alarmingly, all the native sheep populations we examined registered an estimated *Ne* below 500, with none surpassing 250, suggesting potential endangerment. It’s imperative to note that *Ne* estimations are contingent on various factors, including the software employed, sampling methodologies, and analytical techniques. For more reliable results to guide future conservation strategies, we advocate for an extensive, genome-wide *Ne* studies of local sheep breeds. Nevertheless, our observations resonate with the FAO’s classification^25^ that identifies several native European breeds as endangered. And thus, we conclude that, while these sheep breeds show a decline in within-breed genetic diversity, a significant level of genetic diversity still exists. This provides a valuable opportunity for within-breed selection, which could lead to breeding offsprings that are resilient to climate change such as heat stress.

### Inter-population genetic relationship

The analysis conducted through the AMOVA elucidated that the majority of genetic variation (91.06) occurs within populations while only 9% can be attributed to among breeds. This is substantiated by an overall FST value of 0.09 (*p* < 0.001; see Supplementary File 1: Table S2), suggesting low to moderate levels of differentiation among these breeds. These findings closely agree with the global FST value of 0.0869 for goats as reported by Colli et al.^19^. However, it is noteworthy that present Fst value (0.09) is lower than the value obtained for global sheep (0.25;^26^), possibly because, the later study encompassed all types of sheep breeds including commercial breeds.

The first two PCs (Fig. 1) accounted for only 30% of the genetic variation between breeds, indicating a moderate clustering. The most significant, PC1, which explains 17.59% of the genetic variance, distinctly segregates East Africa breeds from their counterparts from North Africa and Asia while European breeds are split in the middle by PC1. Expectedly, genomes of HUN.Dor and HUN.Dor.W correlated with East African breeds (Fig. 1a). PC2 on the other hand effectively differentiates European breeds from their Asian and African counterparts. Differentiating European clusters remained challenging, with a few exceptions like GRC.Chios, LTU.Bface, and certain Swedish breeds that form distinct clusters. The clear distinction between the European clusters, and the Asian and African groups highlights a significant differentiation among these continental sheep populations. This suggests that climatic patterns, in conjunction with geographical distance, may influence their genomic variability.

Moreover, PC3 (Fig. 1b) puts Asian and North African breeds together, likely due to their origin from regions with analogous desert-like climatic conditions. The relationship between breeds based on Reynolds’ distance produced three clusters congruent with the PC1 and PC2 results except for the proximity of BAR and BARL to SWE.Dal and SWE.Fja respectively (Fig. 2).

From the development perspective of breeds, European sheep breeds tend to cluster together, with sub-clusters representing breeds with similar evolutionary history, such as the Pramenka sheep^27,28^ and Nordic breeds. As anticipated, African, and Asian breeds (Except BAR and BARL) form subgroups on one branch, possibly reflecting adaptations to specific climatic conditions or shared developmental histories. The proximity of Greek breeds to African and Asian indigenous breeds is intriguing and could be attributed to adaptations to the Mediterranean climate or shared developmental trajectories. Historically, Greece’s proximity to the Anatolian Peninsula, a cradle of agriculture and early sheep domestication, ensured a continuous interlink between Asia and Africa. This connection was further cemented during Greece’s tenure under the Ottoman Empire, which facilitated the movement of both people and livestock across North Africa, parts of Asia, and Europe.

In summary, our analysis underscores the distinctiveness of African and Asian breeds from their European counterparts, a finding that resonates with studies on global goat populations^19^. However, our results agree with a comprehensive study on global sheep genetics that indicated weak geographical structuring^2,10,26^. This is more pronounced in European breeds, possibly due to the historical events in Europe that occurred several generations ago. For example, Roman empire’s expansion and middle age trade and migration may have contributed to the gene flow between these sheep populations. We also hypothesize that the transboundary and specialized breeds might have influenced the present genetic structure of native sheep breeds. Such breeds often interbreed with indigenous breeds to enhance productivity traits.

The admixture analysis revealed a cross-entropy error at K = 26 out of 33 studied breeds (Supplementary File 2 Figure S1 a), indicating significant admixture. At K = 33 (the number of breeds analyzed), many European sheep breeds, with the exception of LTU.Cwool and SWE.Got, demonstrate high levels of admixture. In contrast, their African and Asian counterparts, excluding KEN.RedM and North African breeds—which exhibit a homogeneous pattern of admixture—form distinct clusters (Fig. 3, detailed analysis of admixture pattern between K2 and K33 is presented in Supplementary File 2 Figure S1 b). This observed level of admixture in European sheep breeds supports the findings of Kijas et al.^26^, which highlighted historical admixture among global sheep breeds. Furthermore, our study reinforces the notion that European Nordic sheep breeds possess unique genomic regions that differentiate them from other indigenous European sheep breeds^29,30^.

### Selection signatures for adaptation

The genetic adaptability of local sheep breeds, which are believed to be well-suited to diverse climatic zones, was examined using two distinct and complementary haplotype methods. RSB assesses the length of haplotypes linked to identical alleles across populations, whereas XP-EHH analyzes the integrated extended haplotype homozygosity between populations. It emphasizes the differences in haplotype structure and frequency among groups, which may signify adaptive genetic alterations. Three demographic population groups were established according to the perceived climate to which individual breeds are adapted. The emphasis was placed on three distinct climatic areas, which are also characterized by their geographical position. The breeds encompass East Africa breeds-adapted to tropical climate, North African-adapted to desert climates, and European breeds- mainly regions experiencing continental climates.

Out of a total of 371 located genes (Supplementary File 1: Tables S3-S8), only 9 (*BMAL1*,* BMPR2*,*CD28*,*CTLA4*,*PRKAG3*,*TUBA4A*,* ATXNL3B*,* RELN*,* KDR*) were common in all the three population pairs. A pair of East Africa vs. European populations had 24 genes in common with the East Africa vs. North Africa pair. Additionally, the East Africa and European pair shared 34 genes with the North Africa vs. European pair. Furthermore, the East Africa and North Africa pair had 45 genes in common with the North Africa and European pair. While we acknowledge the commonality of certain climatic characteristics among these zones, we note that the distinct genes discovered within population pairings indicate the unique adaptation of the examined populations to their separate climatic environments.

Generally, gene interaction network showed that the majority of the genes are functionally related (co-expression ranged between 26.90 for genes located in East Africa vs. European pair and 41.90 for genes located in East Africa vs. North Africa pair. Furthermore, the gene ontology enrichment term of genes identified per population pair indicated dominant gene activities associated with various immunological responses, such as “*Cytokine signaling in the immune system*” and “*Positive regulation of protein modification process*” (see Supplementary File 3, Figures S2–S5). For example, cytokine signaling pathways in sheep have been linked to the immune responses to certain infections, such as those caused by parasites like *Fasciola gigantica* and *Haemonchus contortus*^31,32^. Further, type-1 cytokine response is associated with greater resistance to liver fluke infections, highlighting the important involvement of cytokines in this resistance^31^. On the other hand, the positive control of protein modification processes in sheep is closely connected to their genetic diversity, physiological adaptations to environmental challenges and proteome responses to climate change-induced stresses^33–35^ .

The distinct number of genes identified in each population pair provides evidence that local sheep breeds have experienced differential selection pressure caused by environmental factors. Environmental parameters, including temperature, prevalence of parasites and diseases/vectors, rainfall, etc., vary throughout various climatic zones. These factors may have an impact on selection and shape the genetic architecture of species.

The subsequent discussion emphasizes the importance of certain genes that have been identified in each pair of populations.

### East Africa vs. North Africa population

East Africa consistently has high temperatures throughout the year, along with evenly distributed rainfall. In contrast, North Africa undergoes more pronounced seasonal climate variations, with scorching summers and mild winters. The region is characterized by arid conditions, resulting in low-quality grazing. Gene-gene interaction network analysis revealed a 41% co-expression, indicating the epistatic relationships among the identified genes. Furthermore, the gene ontology enrichment terms demonstrated that the positive regulation of protein modification processes and cytokine signaling in the immune system were over-represented, underscoring the significance of immune response and protein modification in adaptation (see Supplementary File 2, Figure S2 a and b).Supplementary File 1, Table S3 presents the common genes identified by both the RSB and EP-EHH methods in this population pair. The ARF5 gene, located on chromosome 4 in sheep, belongs to the ARFs gene family, which includes ARF1, ARF2, and ARF6. These genes are primarily associated with intracellular localization^36^. However, in certain species, particularly sheep, the ARF5 gene might possess additional functions, such as instigating immune responses and overseeing apoptosis. This is particularly relevant for sheep residing in desert conditions, as they are frequently subjected to extreme temperatures that can induce substantial cellular apoptosis. Other genes that have been postulated to anchor the immune response encompass the CD28 gene and the IL6, IL4, IL13, IL5, CSF2, IL3^37,38^.

While literature offers limited insights into the role of gene DDX2 in sheep, research on pigs indicates its positive regulatory effect on the porcine reproductive and respiratory syndrome virus^39^. In the context of sheep, we hypothesize that this gene might collaborate with genes GLUD1 and BMPR1A on chromosome 25 in modulating reproductive functions^40,41^.

### East Africa vs. European sheep populations

In comparison to Tropical sheep, breeds from Europe, in regions experiencing continental climate have evolved to adeptly navigate the seasonal fluctuations in both temperature and precipitation. Supplementary File 1, Table S4 shows the shared genes found by both the RSB and EP-EHH methodologies in this population pair, whereas Supplementary File 2, Figure S3 a and b illustrates the connection between these common genes and the enriched terms ontology. compared to the preceding population pairs, this pair demonstrated a significant degree of gene co-expression, with biological functions related to immunity being notably over-represented, including cytokine signaling in the immune system, regulation of immune tolerance by vasoactive intestinal peptide, and modulation of mononuclear cell proliferation. Notably, the SLC30A7 gene is instrumental in the translocation of minerals within the organism, with a specific emphasis on Zinc. This mineral is indispensable for a plethora of enzymes that orchestrate growth, reproduction, developmental processes, and immune responses^42^. Consequently, maintaining cellular zinc equilibrium is paramount; a deficiency can culminate in hindered growth, compromised immunity, and reproductive challenges. Furthermore, studies on murine models have associated the SLC30A7 gene with antioxidant stress mitigation^43^. Since continental acclimated sheep should be able to withstand seasonal change stress when the animals are exposed to either an extremely hot summer or a cold winter, the gene may play a significant role in the ability to adapt to continental climate, as compared to tropical acclimated sheep, which are constantly exposed to high temperatures, as well as, poor pasture which also generates certain levels of stress. EGR1 gene is a zinc-finger factor EGR1 that belongs to the early response gene family and is promptly activated by a variety of biological agents, including growth factors, hormones, and neurotransmitters^44^. Since it regulates a variety of biological functions, such as cell differentiation, death, and migration^45^, this gene is considered to be multifunctional. Herein, we suggest that this gene is responsible for both immune response and growth in sheep. ANXA2 gene, on the other hand, is reported to influence milk protein and fatty acid composition^46,47^, while another study depicted ANXA2 gene as being responsible for immune response during acute inflammation^48^. In addition, gene GDI2 has been linked to a number of biological processes, including induction of antibodies against different types of human tumor cells and embryonic development^49^.

### North Africa vs. European populations

The common genes uncovered by both RSB and XP-EHH in this population pair are listed in Supplementary File 1, Table S4. Additionally, the results of gene network analysis and the biological roles associated with the discovered genes are detailed in Supplementary File S3, Figures S4a and b. Similar to the first population pair, the positive regulation of protein modification processes and the management of immunological tolerance by vasoactive intestinal peptides were among the biological functions that were overrepresented by these genes. In contrast to the previously discussed population pairs, gene-network analysis revealed that these genes exhibit a high percentage of physical interaction, indicating that the differential adaptive capacity between the two populations is primarily attributed to direct gene-gene interactions rather than co-expression.

Notably, several genes identified within this climate-based population pair have been postulated to play pivotal roles in the immune response. Genes CXCL1 and CXCL10 have been previously implicated in pregnancy immunity^50^ while CR2 has been implicated in heat stress tolerance^51,52^.

Reproductive efficiency, being a cornerstone of survival across all biotic entities, is of tantamount significance in the context of climate change. Within the purview of our research, we have mapped several genes that underpin reproductive efficiency, notably the multiple ovulation gene INHA^53^. RELN is a protein coding gene that has been linked to brain development and neural migration in many animals and in humans as well^54^. The gene could be vital in adaptation, since adaptation traits are multifactorial and need effective physiological system coordination. Genes like MTNR1B have been linked to seasonal reproduction ability^55^, while others like MSTN were reported to affect growth and muscle development^56^. MSTN suppresses muscle development and its presence in desert sheep is justified since their smaller body size is crucial for heat stress adaptation, as well as, for adaptation to limited feed resources.

### Common genes in all population pairs

For the consistent and enduring provision of animal proteins, the adaptation to environmental factors brought about by climate change is an indispensable trait sought in all livestock species. Given their widespread distribution and remarkable phenotypic diversity, indigenous sheep breeds have been recognized as pivotal model organisms in the exploration of adaptive mechanisms. In the present study, every climatic population pairing yielded unique genes, postulated to be instrumental in facilitating the adaptation of these native sheep breeds to their local environments. Notably, only 9 genes were common in all three population pairs (Supplementary File 1, Table S5). The gene interaction network analysis showed that majority (55.44%) of the common genes interactions are based on biological pathways while 24.96% of the interactions are based on co-expression. The gene ontology enrichment analysis revealed a considerable over-representation of protein phosphorylation function among the nine common genes. Protein phosphorylation plays a vital role in regulating several physiological processes in sheep, such as muscle function, wool fiber formation, and vascular contractility Supplementary File S3, Figures S5 a and b. For instance, Chen et al.^57^ discovered crucial protein kinases, including glycogen synthase kinase 3, cyclin-dependent kinase 5, and protein kinase C, that play a significant role in controlling phosphorylation throughout the growth of wool fibers in Tan sheep. In addition, sheep-derived antibodies have been used to identify phosphorylation sites in skeletal muscles^58,59^, further highlighting the significance of phosphorylation in muscle control.

## Conclusions

In this comprehensive investigation, we elucidated the in depth picture of the genetic diversity and relationship among native sheep breeds, and their adaptability to different climatic zones. Although the breeds under study exhibited a high within breed genetic diversity levels, there is an alarming decline in this diversity, as evidenced by the declining effective population size. African and Asian breeds distinctly cluster themselves, without any overlap, signifying their unique genomic structures. Conversely, European indigenous sheep breeds display a rich genetic admixture, compromising their genetic distinctiveness. We identified over 371 genes that are believed to be under selection in different climatic zones. Intriguingly, the majority of these genes are exclusive to specific climate-based population pairs, underscoring the unique adaptive capacities inherent to each studied group. Notably, gene ontology enrichment showed that *Cytokine signaling in the immune system*” and “*Positive regulation of protein modification process*” pathways were over-represented highlighting the significance of immune responses and protein modification to adaptation and thus emphasizing the imperative of breeding disease-resistant livestock in the current context of climate change. This study reinforces the notion that while indigenous sheep breeds retain substantial genetic diversity, it is diminishing, potentially due to sub-optimal genetic management or admixture events. Furthermore, our findings accentuate the innate capability of native sheep breeds to endure climate change effects, positioning them as invaluable resources for breeding traits associated with climate change resilience, thereby enhancing the overall robustness of sheep populations.

## Methods

### Sample collection and genotyping

In the present investigation, a comprehensive analysis was conducted on 890 samples spanning 33 distinct breeds. Specifically, 620 samples, representing 22 breeds, were freshly genotyped for the purposes of this research. The remaining samples were sourced from publicly accessible databases, as delineated in Table 1. Supplementary File 4: Figures S6 to S27 offers a brief phonotypic description of the breeds from which fresh samples were procured. Furthermore, Supplementary File 1: Table S1 indicates specifics pertaining to the samples from each population, encompassing their geographical source and the inferred climatic conditions of their adaptation.

To preserve the integrity of genetic diversity within each breed, care was taken to ensure that samples were collected from genetically distinct individuals. In the absence of records, we relied on information from breeders to identify unrelated individuals for sampling. The sampling procedure involved puncturing the jugular veins of individual animals to extract blood samples. These samples were subsequently stored in vacutainer tubes and later transferred onto Flinders Technology Associates (FTA) cards^®^. Prior to dispatching to the genotyping facility, the dried-out FTA cards were preserved in ambient room conditions.

The whole processes of DNA extraction, genotyping, and the transformation of raw signals into the Ovine50K genotype were entrusted to Neogen Company Limited. The company employed the Infinium assay protocol for these procedures. Genotypic determinations were updated using the ARS-UI_Ramb_v2.0^60^, which served as the reference genome. Subsequently, a comprehensive report was compiled based on these findings.

**Table 1:** Geographic origins of samples and data sources.

Country/region	Continent	Number of breeds	Number of samples pre-QC	Source of data
Kenya	East Africa	2	90	This study
Ethiopia	East Africa	1	34	Kijas et al.^10^
Morocco	North Africa	Assorted	34	Collaborator
Algeria	North Africa	1	22	Jamaa et al. (2019)
Libya	North Africa	1	13	Kijas et al.^10^
Iran	Southwestern Asia	2	71	Kijas et al.^10^
China	East Asia	1	37	Kijas et al.^10^
Hungary	Central Europe	8	200	This study
Romania	Southeastern Europe	2	55	This study
Greece	Southeastern Europe	2	62	This study
Poland	Central Europe	2	50	This study
Serbia	Southeastern Europe	2	45	This study
Bosnia and Herzegovina	Southeastern Europe	3	83	This study
Lithuania	Northern Europe	1	30	This study
Sweden	Northern Europe	4	64	Rochus et al.^14^

Assorted: individuals came from different breeds; collaborator: the researcher who shared with us his genotyped data, it signifies that the data used in our study were neither genotyped for this study nor obtained by downloading, but rather were provided to us by our collaborator; Number of samples pre-QC; the initial number of samples included in the study before quality control.

### Post genotyping data management and SNP quality control

Upon receipt of the fresh genotyped data encompassing 620 genotypes from Neogen, the data was meticulously transformed into PLINK binary files^61^. Subsequently, these files were merged with previously acquired datasets, yielding a comprehensive dataset comprising 890 samples and 83,533 SNPs. Quality control measures were stringently enforced based on the ensuing criteria:

1. Exclusion of closely related or duplicate samples.
2. Omission of SNPs located on sex chromosomes and those of indeterminate origin.
3. Removal of samples with over 10% missing data.
4. Removal of SNPs with over 10% missing genotypes.
5. Exclusion of SNPs with a minor allele frequency (MAF) below 0.05%.
6. SNPs deviating from the Hardy-Weinberg equilibrium with a threshold (HWE) of *p* < 10^− 3^ were also excluded.

Post-quality control dataset encompassed 835 samples and 35,949 polymorphic SNPs, poised for further analytical procedures. This curated dataset will be subsequently denoted as the “working dataset.”

### Statistical analysis

In general, three breeds, namely HUN.IldeFr (of French ancestry), HUN.Suff (originating from the British Isles) and HUN.Tetra (a composite breed), were considered as outgroups, since they are not native to Hungary. The primary objective of the statistical analysis conducted in this study was to assess the genetic diversity present within and between indigenous sheep breeds. Additionally, the analysis aimed to investigate inter-breed genetic relationships and identify potential selection sweeps that underpins unique adaptation of native sheep breeds.

### Intra-population genetic diversity analysis

Genetic diversity within and among breeds (populations) was done on all 33 breeds under investigation. To ensure reliable results of genetic diversity, the working dataset was further meticulously refined. This involved the removal of SNPs exhibiting linkage disequilibrium (LD) to minimize the potential of results being overly influenced by limited genomic regions. Specifically, the LD-pruning procedure was implemented to exclude SNPs until no two SNPs exhibited a correlation coefficient (r^2^) exceeding 0.5 within a span of 200 kb. Subsequent to this refinement, standard genetic diversity metrics were ascertained utilizing the Arlequin software, version 3.5^62^. These metrics encompassed both expected and observed heterozygosity (He and Ho), which were derived from haplotype frequencies. Additionally, the degree of inbreeding (F_HOM_) as proportion of successfully genotyped loci which were homozygous^63^. The SNeP software^64^ was utilized to evaluate the past patterns in effective population size (Ne), employing the default configurations. Due to the reliability of 50 K SNP chips in measuring effective population size within a maximum of 50 generations^5^, we only obtained data for effective population sizes at 13 and 45 generations back. In order to enhance precision, we harmonized breed sample size to between 16 and 20, and those with more samples were randomly resampled using “slice_sample” function implemented in tidyverse, to 20 individuals.

### Inter-breed genetic relationship

To determine the genomic variation within and between populations, the analysis of molecular variance (AMOVA) of sheep populations was examined using the Arlequin software version 3.5^62^. AMOVA was performed on all 33 breeds using distance matrix based on pairwise differences.

In order to ascertain the genetic positioning of different local sheep breeds in relation to each other using principal components (PCs), and to assess if the sampled animals were from a genetically homogenous population, a principal component analysis (PCA) was conducted using plink 1.9^61^. Neighbor joining tree was developed based on between-breed Reynolds distance which were calculated by Arlequin software.

Also, to determine the population differentiation from each other, Wright’s Fixation index (FST) was computed on primary dataset using the R package StAMPP^65^ to produce the FST matrix with their significance. To quantify population structure and individual ancestry sources of the primary samples, we employed ADMIXTURE v1.3.0^66^. The study was carried out using the priori determined ancestry clusters (K), which were assumed to represent a range of 2 to 35 ancestral populations. The 15-fold cross validation error with the lowest value was used to select the most probable K. The Q matrix generated was visualized using BITE package v2^67^.

### Identification of genomic signatures for adaptation

In this part of the analysis, the SNP dataset utilized was neither filtered for minor allele frequencies (MAFs) nor pruned for pairwise linkage disequilibrium. This analysis focused only on indigenous breeds, hence Hungarian breeds that were used as outgroups were not included. Furthermore, our primary objective was to assess the genetic variation in adaptability among tropical, continental, and North African desert conditions. To discern genomic markers indicative of adaptation to varying environmental conditions, we undertook a comprehensive pairwise comparison of populations, categorized based on their climatic/geographic origins. This approach was strategically chosen to minimize biases that might arise from uncharacterized population structures. For the purpose of this study, populations were classified into three distinct groups:

1. Tropical climate/ East African group: This consisted solely of breeds originating from East Africa, specifically Ethiopia and Kenya.
2. North African/Desert climate: This group comprised breeds from the North African regions, notably MAR and Algeria and Libya.
3. Continetal climate group: This category included indigenous sheep breeds from Europe, with the exception of breeds from Sweden, Lithuania, HUN.Dor, HUN.Dor.W, and the designated outgroups.

Consequently, any breeds that do not belong to these groupings were excluded. The group membership was standardized to 109, which matched the number of samples in the smallest group. To identify potential signatures of adaptation, we employed two complementary robust haplotype based methods; the Cross Population Extended Haplotype Homozygosity (XP-EHH) method and, the Ratio of extended haplotype score between populations (Rbs;^68^). Outliers SNPs were identified following the method described by Gautier^68^ which involves which involves genome-wide scanning using sliding windows. SNPs were classified as “candidates” if they met the specified criteria, including a minimum of one marker per window, at least two extreme markers, a minimum percentage of extreme markers among all markers, and a sliding window size of 0.1^− 6^ bp. The BioMart package was utilized to identify protein-coding genes located in proximity to genomic regions recognized as signatures of selection. Gene annotation was performed with the ARS-UI_Ramb_v2.0 annotated genome^60^ for both the positive and negative strands.

## Electronic supplementary material

Below is the link to the electronic supplementary material.

Supplementary Material 1

Supplementary Material 2

Supplementary Material 3

Supplementary Material 4

Supplementary Material 5

## Data Availability

The Fresh genotyped underlying this article are available in the Figshare, at 10.6084/m9.figshare.24321127.
