# Supplementary material for: Genetic diversity and adaptability of native sheep breeds from different climatic zones
Source: Sci Rep. 2025 Apr 23;15:14143. doi: 10.1038/s41598-025-97931-2 (PMC12019589; doi:10.1038/s41598-025-97931-2)
Supplement: Supplementary file 2 — Supplementary Material 2 [file 41598_2025_97931_MOESM2_ESM.pdf]

Supplementary file 3: Figures S2-S5

Gene-gene interaction network for genes identified in East Africa vs North Africa native sheep breeds

(a)

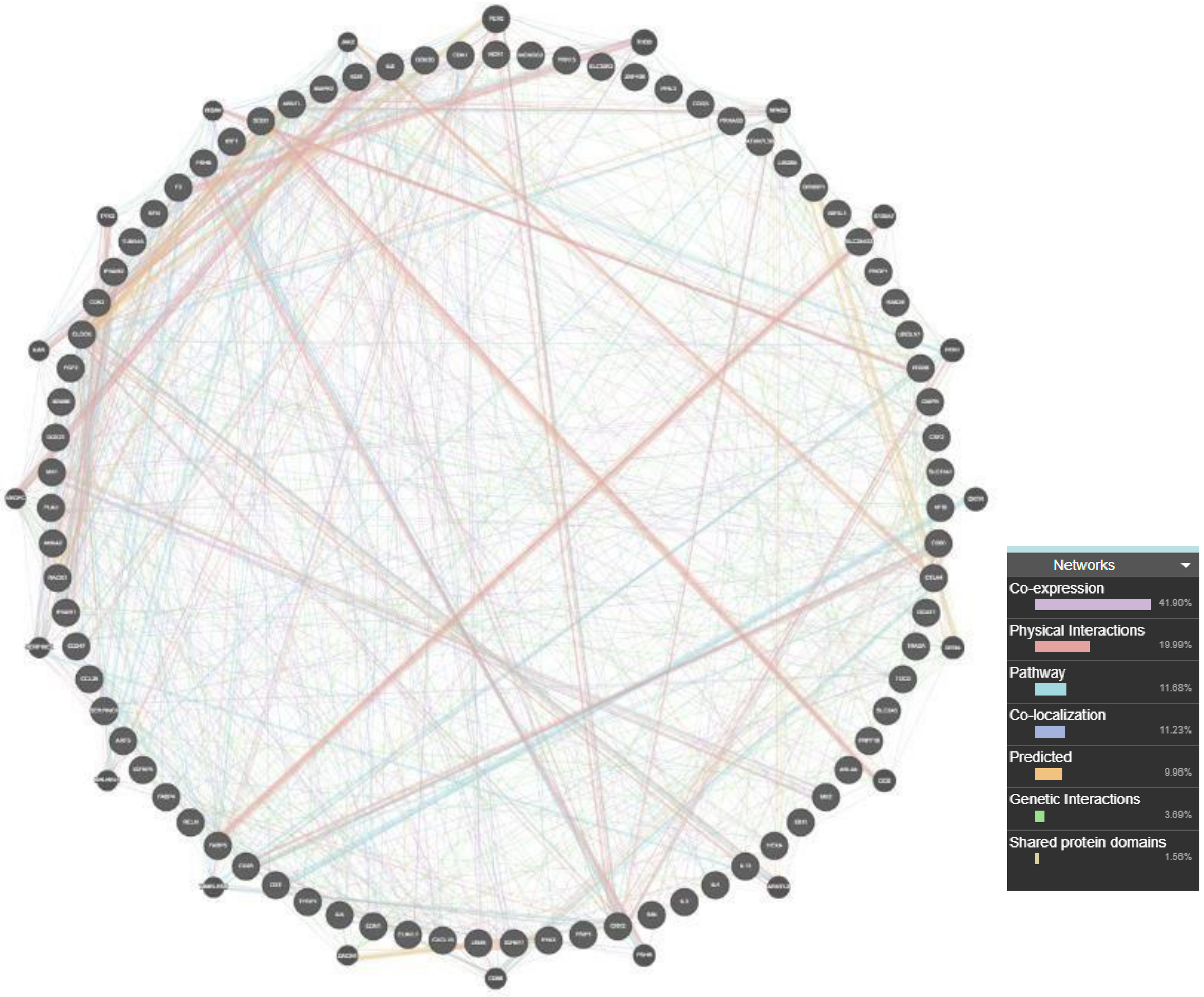

(b)

A heatmap of Gene-ontology enrichment for genes identified in East Africa vs North Africa sheep breeds

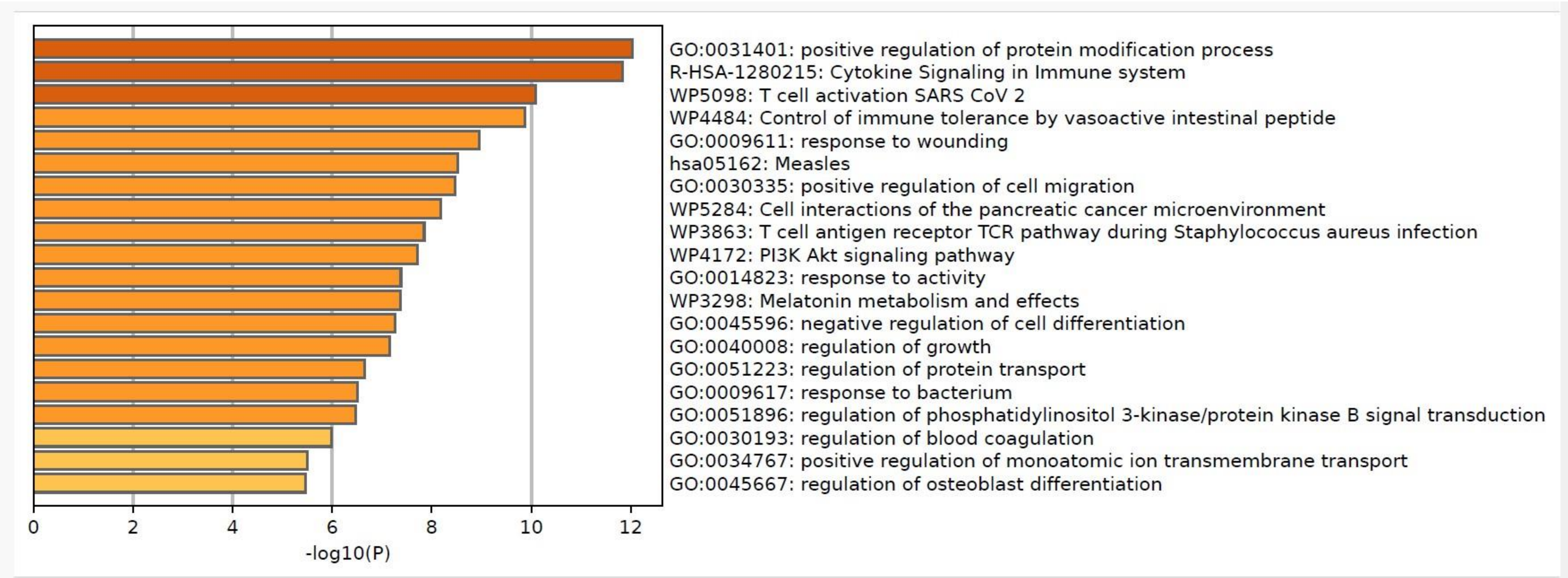

Figure S2: Gene interaction network and enrichment terms for genes identified in East Africa vs North Africa sheep breeds

Gene-gene interaction network for genes identified in East Africa vs European native sheep breeds

(a)

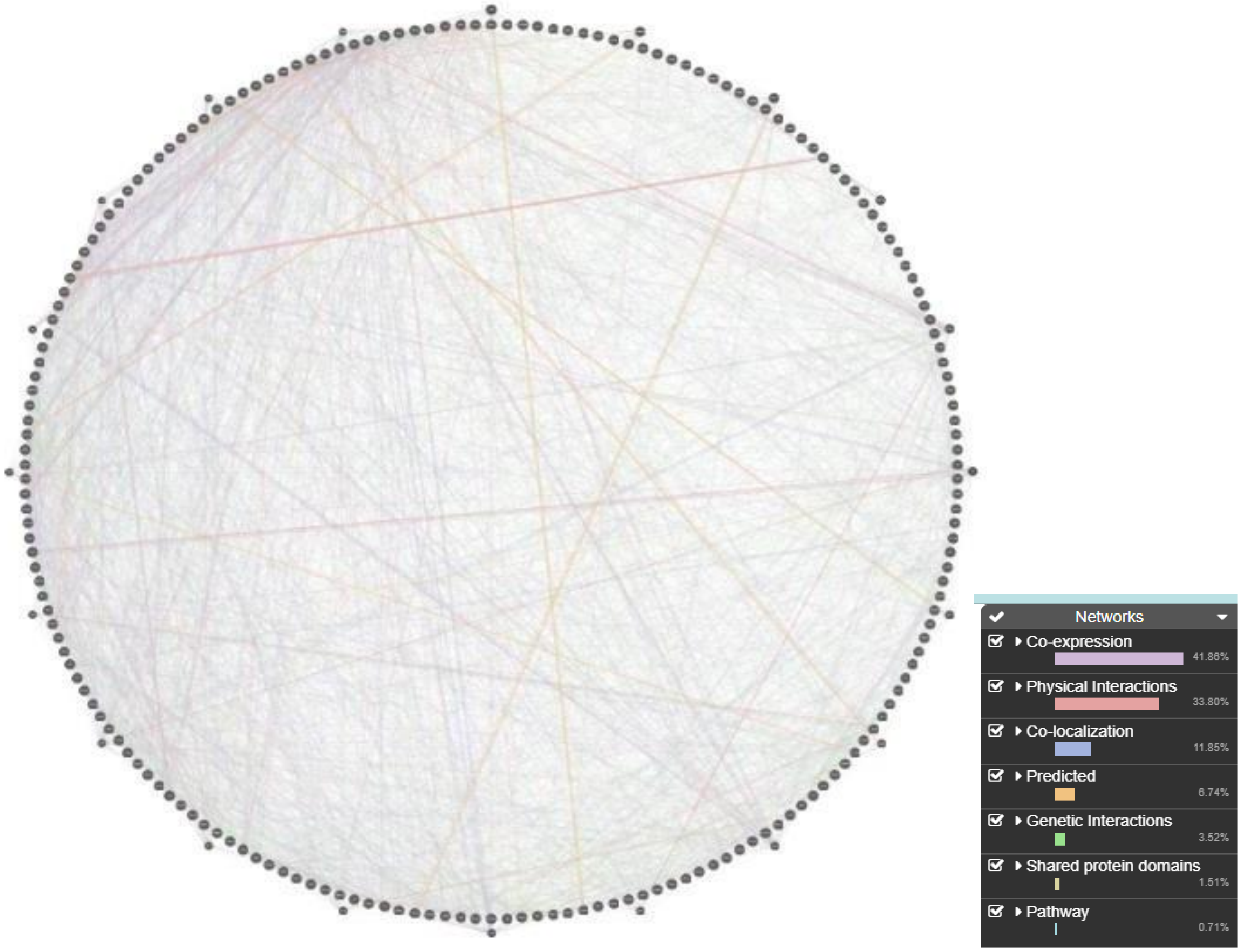

A heatmap of Gene-ontology enrichment for genes identified in East Africa vs European sheep breeds

(b)

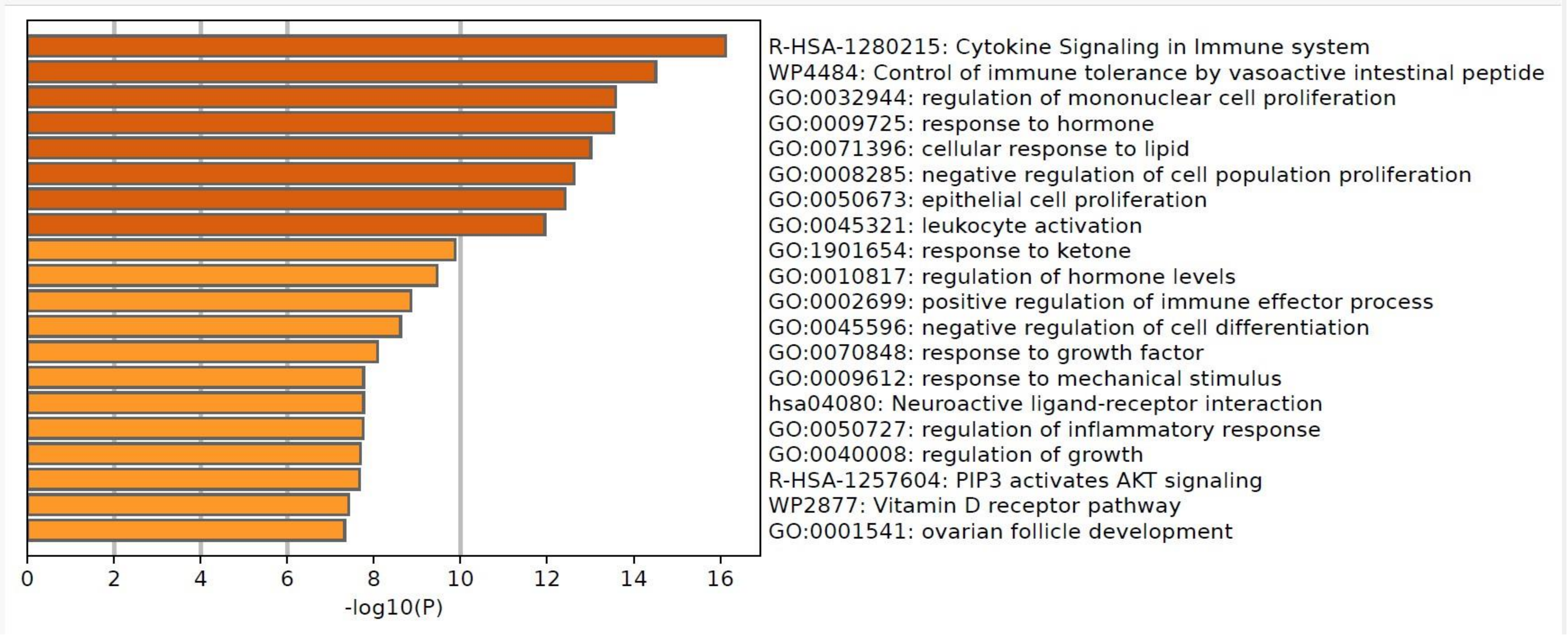

Figure S3: Gene interaction network and enrichment terms for genes identified in East Africa vs European sheep breeds

Gene-gene interaction network for genes identified in North Africa vs European native sheep breeds

(a)

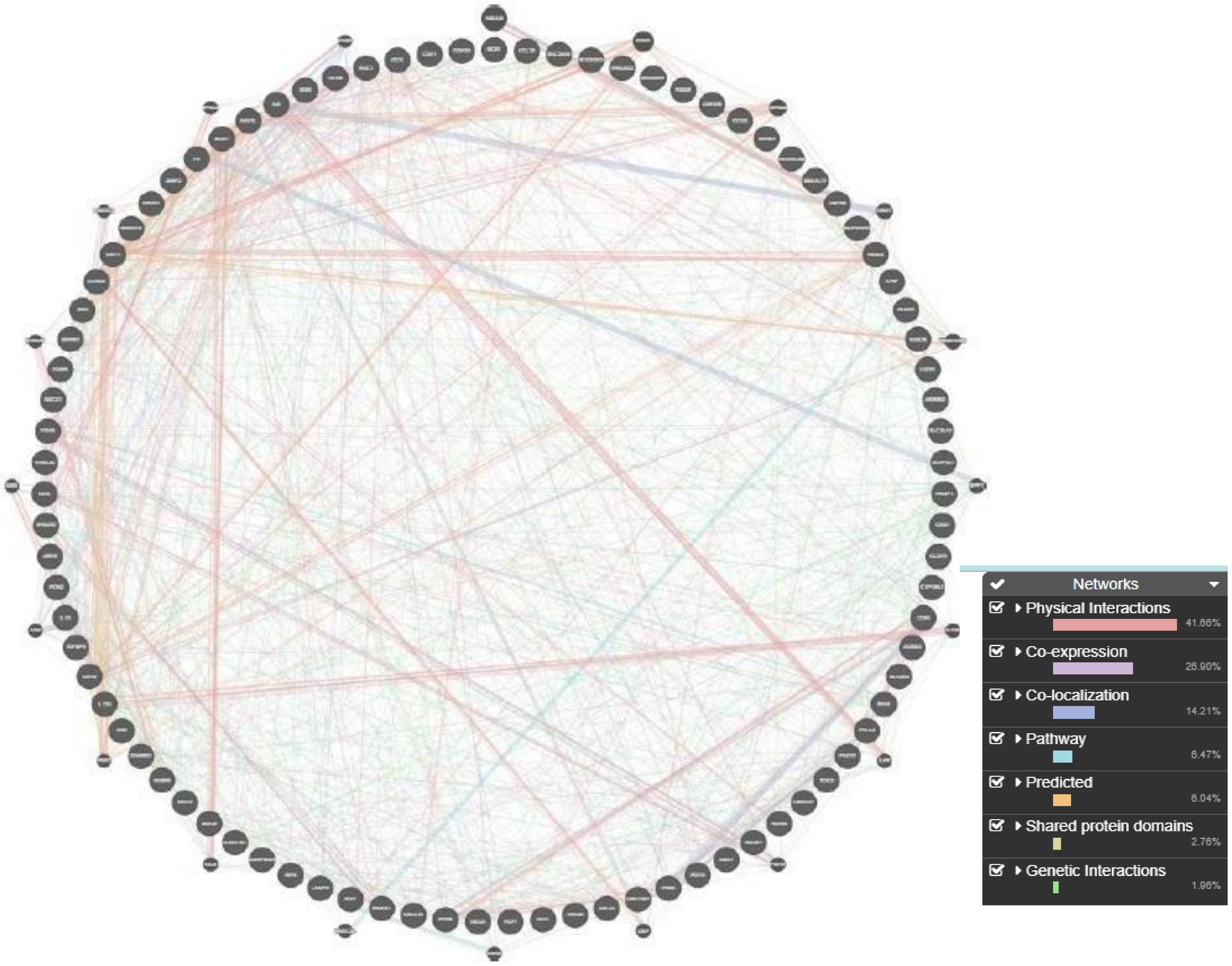

A heatmap of Gene-ontology enrichment for genes identified in North Africa vs European native sheep breeds

(b)

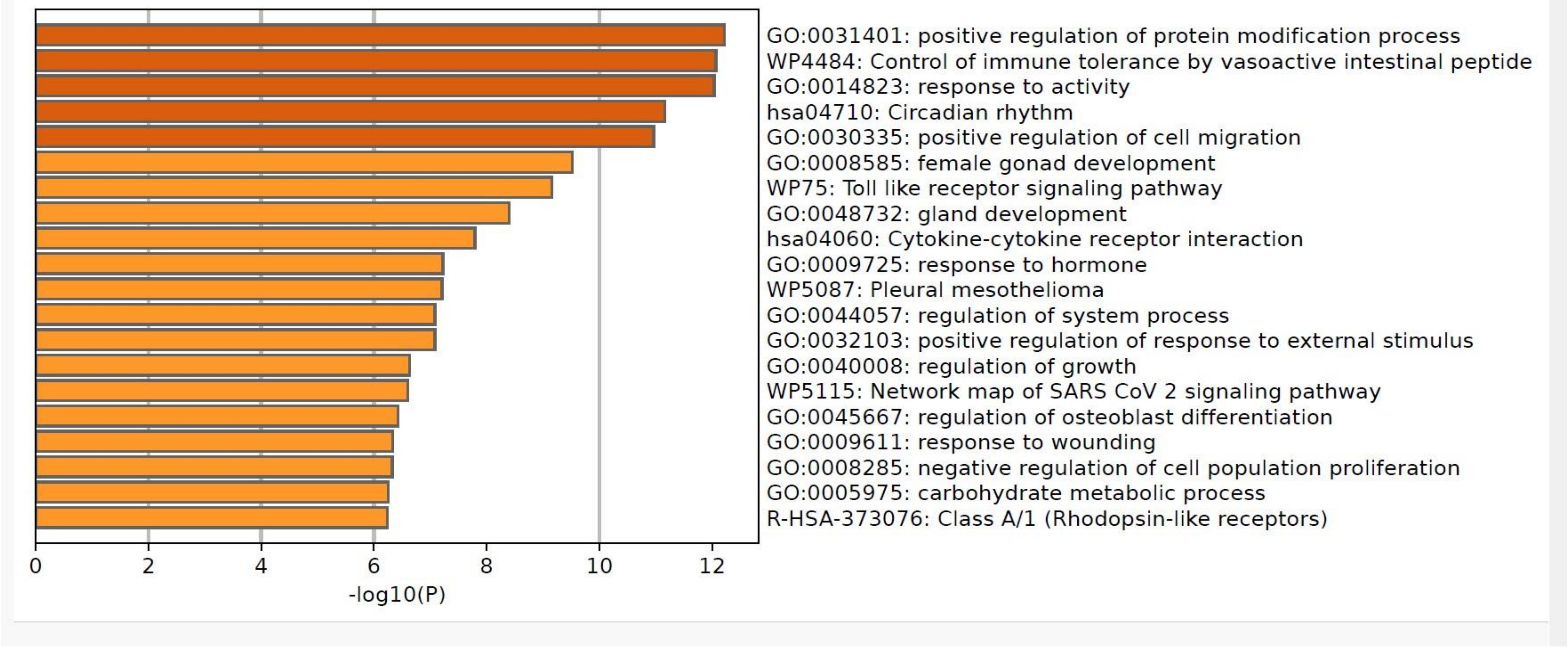

Figure S4: Gene interaction network and enrichment terms for genes identified in North Africa vs European sheep breeds

Gene-gene interaction network for common genes identified in all population pairs

(a)

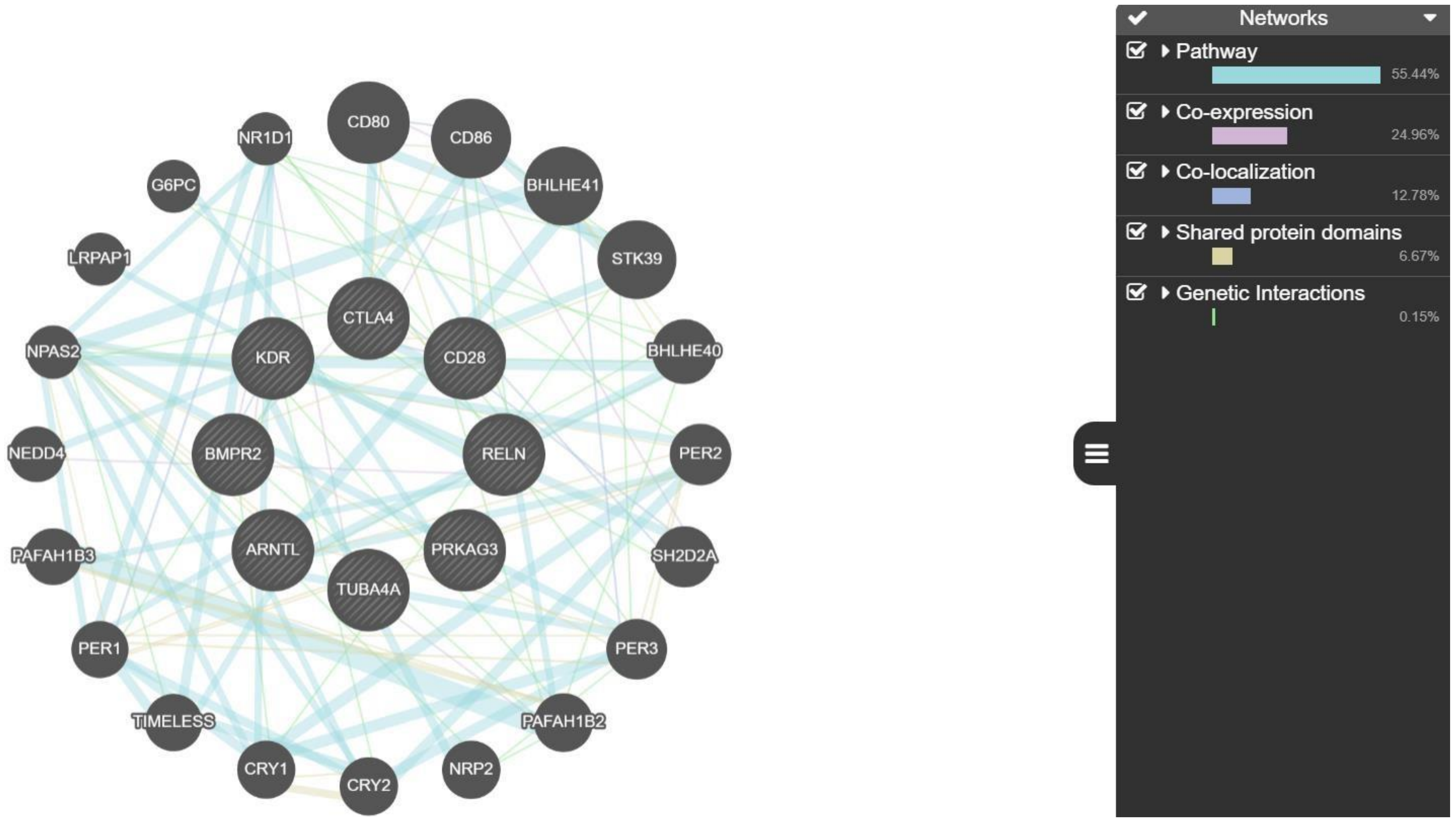

A heatmap of Gene-ontology enrichment for common genes identified in all population pairs

(b)

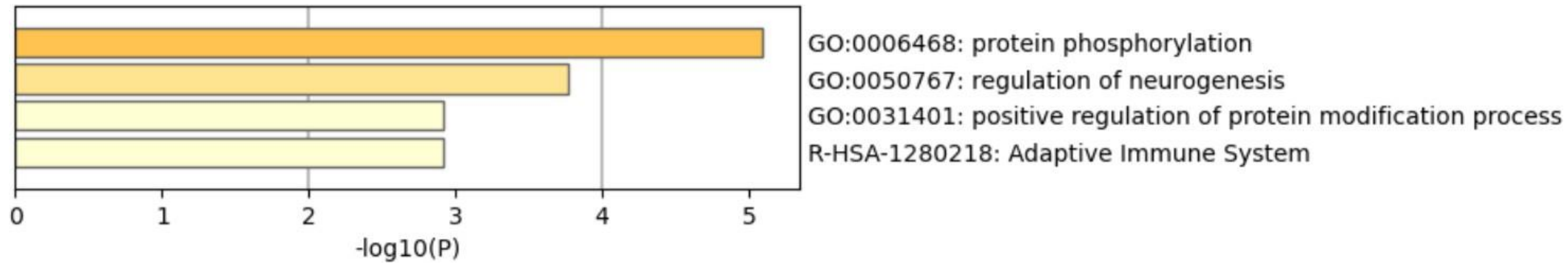

Figure S5: Gene interaction network and enrichment terms for common genes identified in all population pairs
