# Supplementary material for: Genetic diversity and adaptability of native sheep breeds from different climatic zones
Source: Sci Rep. 2025 Apr 23;15:14143. doi: 10.1038/s41598-025-97931-2 (PMC12019589; doi:10.1038/s41598-025-97931-2)
Supplement: Supplementary file 4 — Supplementary Material 4 [file 41598_2025_97931_MOESM4_ESM.pdf]

Supplementary file 3: Breed description

Polish breeds

Heath Sheep (Polish Wrzosówka)

Heath Sheep (in polish Wrzosówka) is a Polish native, old breed of sheep, belonging to the group of Northern short-tailed sheep, found in Northern Europe. Heath Sheep are small sheep with a gray mixed wool coat; lambs are born black and turn gray as they age. The Heath Sheep is a sheepskin breed; produces excellent quality leather. It is characterized by high fertility and seasonality of reproduction.

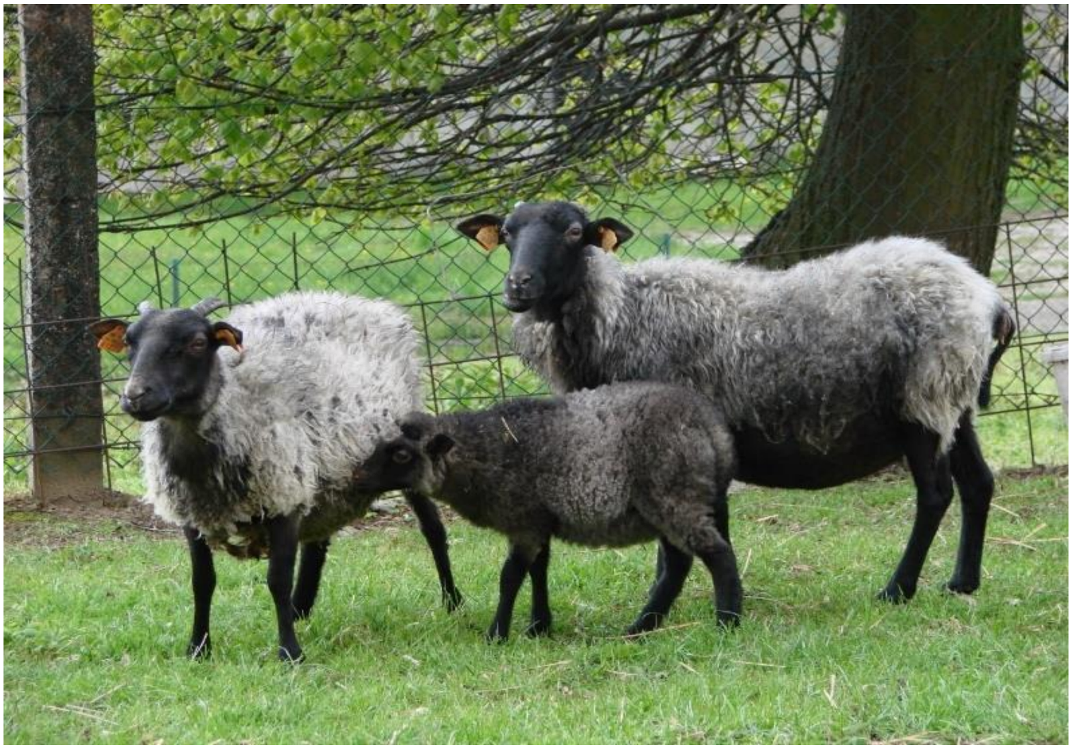

Figure S6: Heath Sheep
Photo by Aldona Kawęcka

Podhale Zackel (Cakiel podhalański)

Podhale Zackel (in Polish Cakiel podhalański) is a versatile old Polish breed, derived from sheep that came to the Polish Carpathians centuries ago with the Wallachian population. Sheep belong to the group of mountain sheep, they are perfectly adapted to the harsh conditions of the mountains, resistant to diseases, long-lived, with a strong maternal and herding instinct. The woolly coat is white, thick, mixed, two-fraction, dense, with a flock structure.

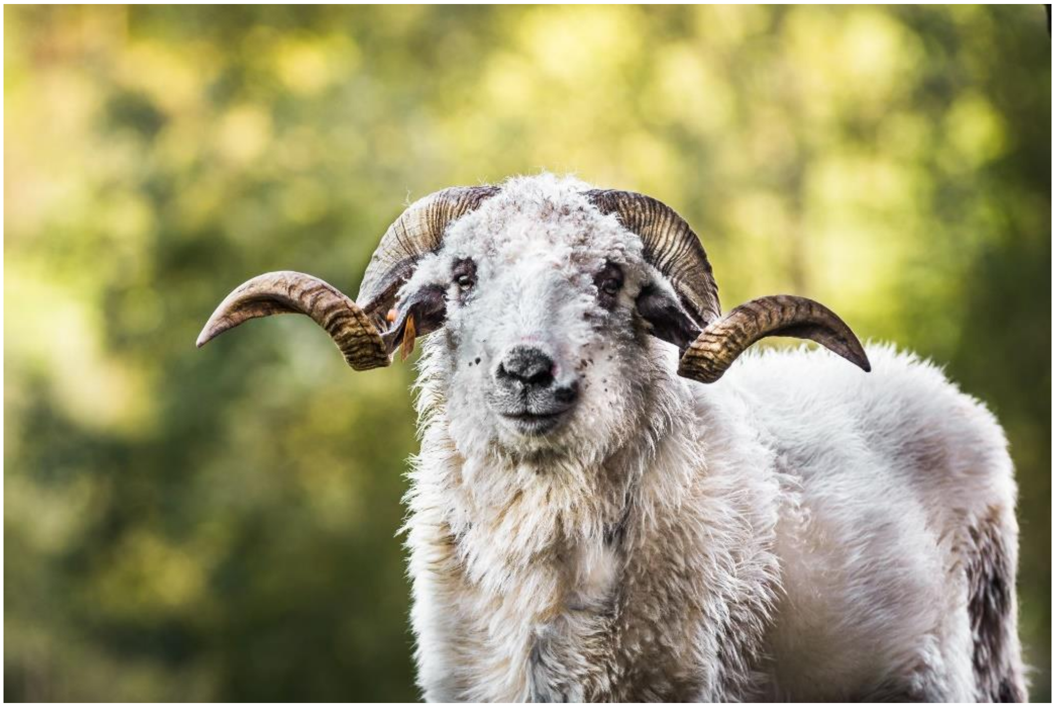

Figure S7: Podhale Zackel
photo by Marta Pasternak

Romanian breeds

White-headed Turcana (Turcana breed)

Turcana is a mountain hardy breed, belonging to the Eastern European Zackel group, originating in the Carpathian Mountains, and having a current census of 7.8 million breeding ewes. The Turcana is traditionally used as a triple-purpose breed, reared for meat (gains of 110-180 g/day), milk (60-150 kg/lactation) and wool (typical coarse long staples, of 20-25 cm). Phenotypically, the Turcana breed is diverse, having five recognized ecotypes, and three colour varieties (white, black and grey), with weights of adult ewes ranging from 30 to 55 kg, and 60 to 110 kg in rams. Turcana rams have long spiralled horns, while ewes can either be pooled or horned. Conception rates in ewes range from 95% to 98%, with the prolificacy varying from 105% to 130%, with extremely good survival rates of lambs, longevity in adult animals and adaptation to cold temperatures, low quality feeding regimes and resistance transhumance and long roads.

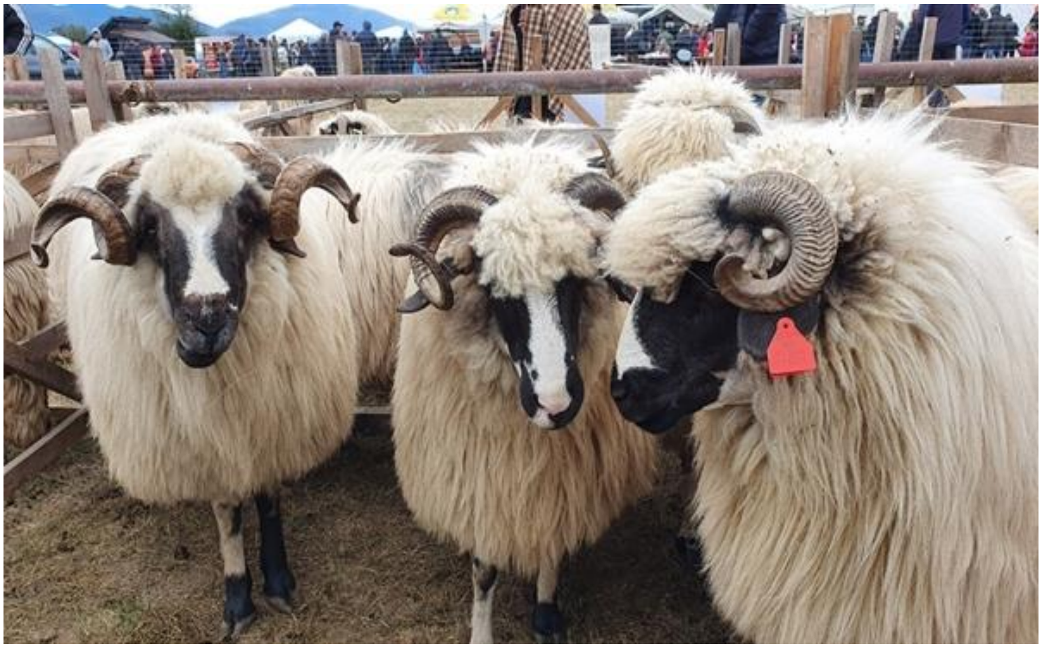

Figure S8: White-headed Turcana
Photo by Vasile Calin Ilisiu

Rusty Tsigai (Romanian Tsigai)

Rusty Tsigaiis an ancient low-land breed, originating in Eastern Romania, with first archaeological proofs of its existence dating over 2000 years ago, having a census of 2.2 million heads. Tsigai sheep are regarded as one of the two main sheep breed groups of Eastern Europe, alongside Zackel group. The Tsigai is traditionally triple purpose, reared for meat (gains of 160-250 g/day), milk (70-110 kg/lactation) and wool (semi-fine staple of 8-10 cm, 3.5-4.5 kg, 30-35 µm). Phenotypically, Tsigai are extremely diverse, weights of adult ewes ranging from 35 to 70 kg, and 55 to 90 kg in rams, most frequent colour varieties being white-headed, black-headed, speckled, and rusty. Tsigai sheep can be pooled or have spiralled horns. In Romania the Tsigai contributed to the formation of local Merino derived breeds. Conception rates in ewes range from 92% to 97%, with the prolificacy varying from 120% to 150% .

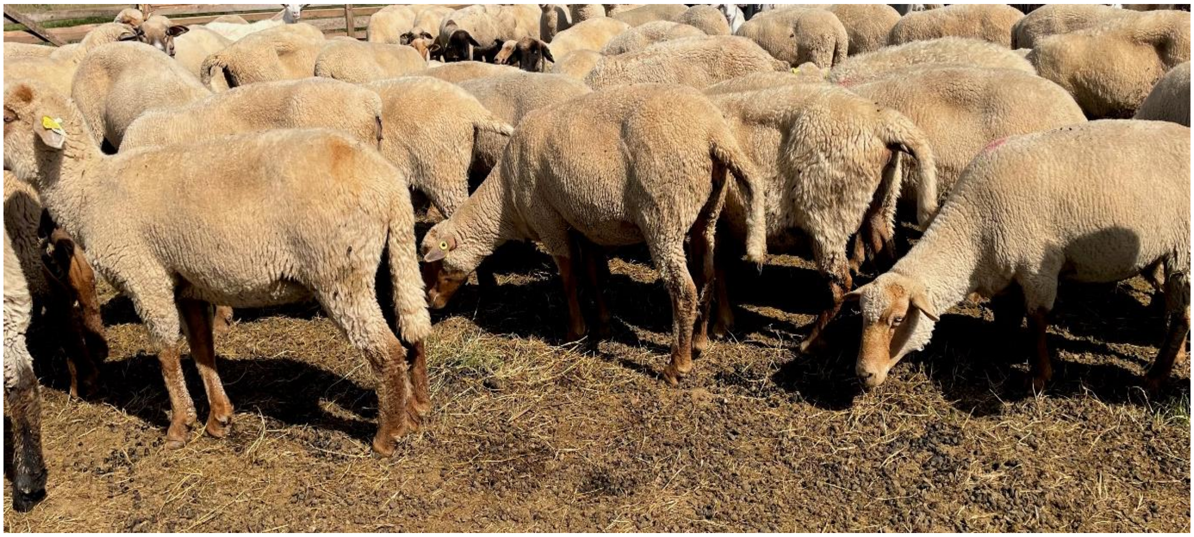

Figure S9: Rusty Tsigai
Photo by Zoltán Bagi

Greek breeds

Boutsiko breed

The Boutsiko breed is classified among the Zackel group of mountain sheep and is mostly distributed over the mountainous regions of Epirus and West Macedonia. The breed's population is estimated to be around 15,000 individuals. The breed has been selectively bred from the Vlahiko, Karakachan, and Grammoustiano indigenous Greek breeds, with the purpose of enhancing milk and lamb production. It exhibits remarkable adaptations to challenging environmental conditions, including steep topography, moderate precipitation, and elevated humidity levels. Generally, this breed is characterized by small size, with females weighing between 35 and 45 kg and males weighing between 45 and 50 kg. The majority of the ewes lamb in November, and their fertility is high (>90%) while the prolificacy rate is low (1.1–1.3). The average milk production is about 100 kg in 180 days of lactation, with a fat content of about 7–7.5%. The carcass weight is about 8–9 kg, with very good quality.

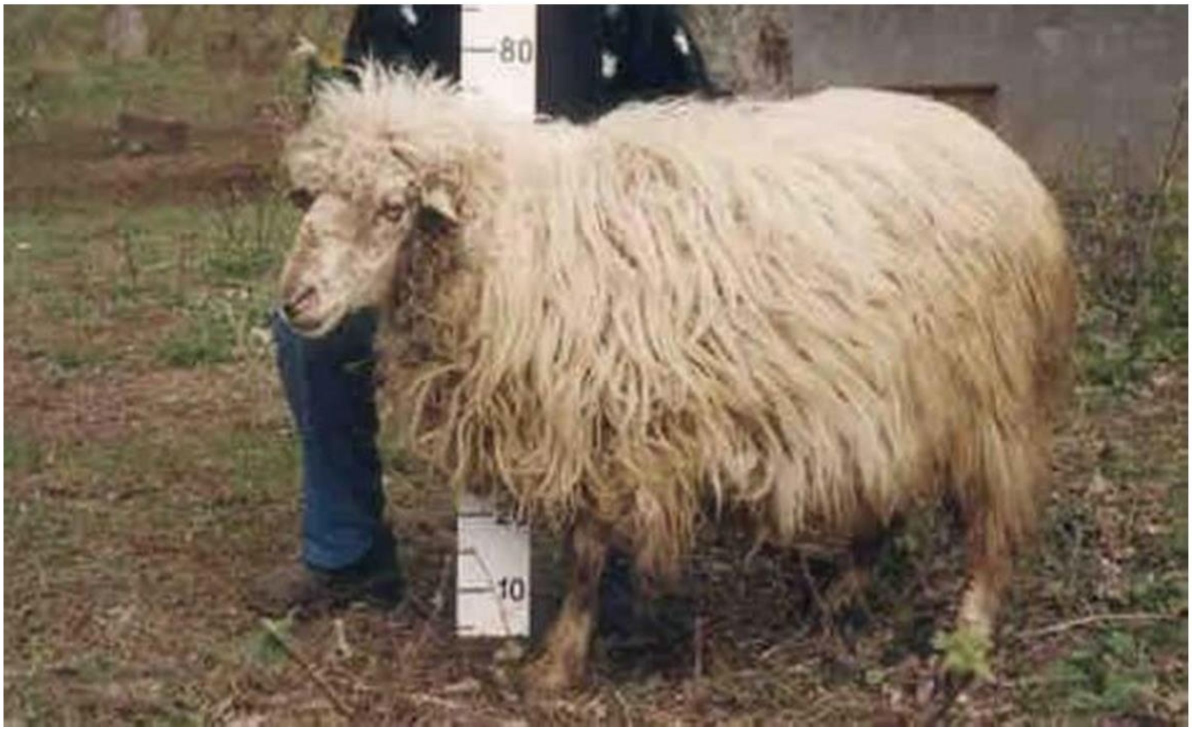

Figure S10: Boutsiko
Photo by Dimitrios Loukovitis

Chios

Chios sheep breed originates from the Greek island of Chios and is classified as a semi-fat tailed breed. It is suggested that the breed is the result of crossbreeding between local sheep of Chios Island and breeds from West Anatolia. Chios sheep are bred mainly for their milk production in semi-intensive farming systems, with a total population of 25.000.

Chios sheep are typically white with black, occasionally brown, spots around the eyes, and on the ears, nose, belly and legs. The entire head is often black. Mature ewes have a wither height between 70 and 76 cm and weigh 50 to 70 kg, while mature rams weigh 65 to 95 kg with a wither height from 79 to 84 cm. Ewes can reproduce twice per year with an average litter size between 1.7-2.2. Milk production of the breed is high, varying from 200 to 320 kg of milk per lactation, depending on management and husbandry conditions. The average lactation period is 210 days and the highest milk production ever recorded is 700 kg in a 220 day lactation period. The milk is used to produce many types of traditional Greek cheeses.

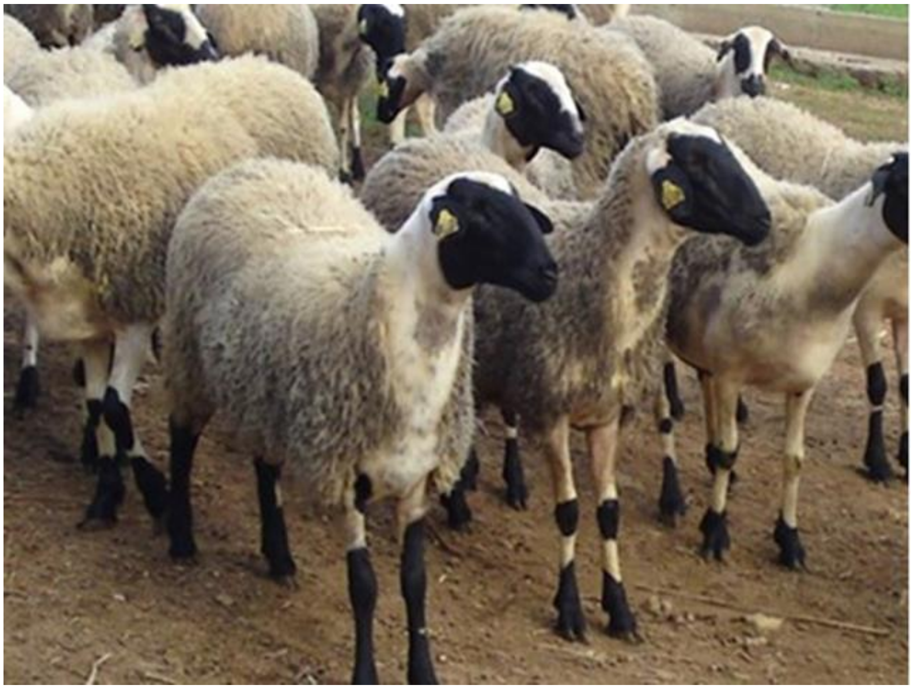

Figure S11: Chios
Photo by Dimitrios Loukovitis

Lithuanian sheep breed

Lithuanian Coarse wool sheep

The Lithuanian Coarse Wool sheep is classified as a medium-sized breed, characterized by a mature ewe weighing between 30 to 40 kilograms, while a mature ram typically weighs between 40 and 50 kg respectively. This breed possesses a long, rough, and fibrous fleece, often exhibiting a white hue, but variations in coloration such as black, brown, or gray are also seen. Their wool is used in the production of customary Lithuanian textiles, including rugs, blankets, and garments. The sheep breed has slender limbs, often exposing a bare abdomen adorned only with coarse fleece, a robust dermis, a small nose, and a broad face. In addition, their ears are relatively small with a moderate length. This breed is highly regarded as a cold resilient breed as well as resistant to various types of diseases and parasites. Their grazing habits are commendable although they have a tendency of low maturity., but with delayed maturation. The Lithuanian Coarse Wool sheep breed is a docile breed which is classified as a dual-purpose, as it produces both meat and wool. The yearly fleece weight ranges from 1.0 to 1.5 kg and consists of wool in various colors such as grey, white, tan, and black. The breed is also highly prolific; ewes produce about 2 to 3 lambs per lambing season.

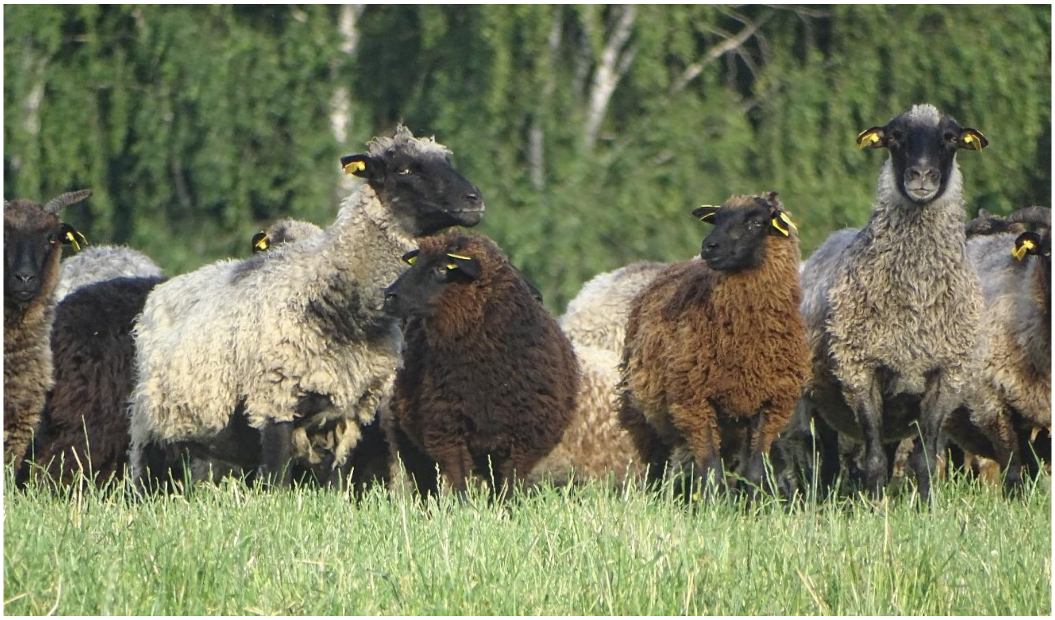

Figure S12: Lithuanian Coarse wool sheep
Photo by Ruta Sveistiene

Kenyan sheep breeds

Red Maasai

The Red Maasai is a fat-tailed breed that is native to the East African region. It mainly produces meat and occasionally milk. The breed is highly regarded among pastoral communities due to its resilience in dry environments and the ability to resist internal parasites, particularly *Haemonchus spp*. The Red Maasai sheep breed has red or brown hair. Their tail is long and fat, while their ears have upright and elongated characteristics. On average, adult females weigh 50 kg, while males weigh 70 kg. Red Maasai are characteristically docile, with early reproductive maturity. Ewes generally start their first lambing at around 18 months of age, and they possess the capability to undergo two lambing seasons within a year.

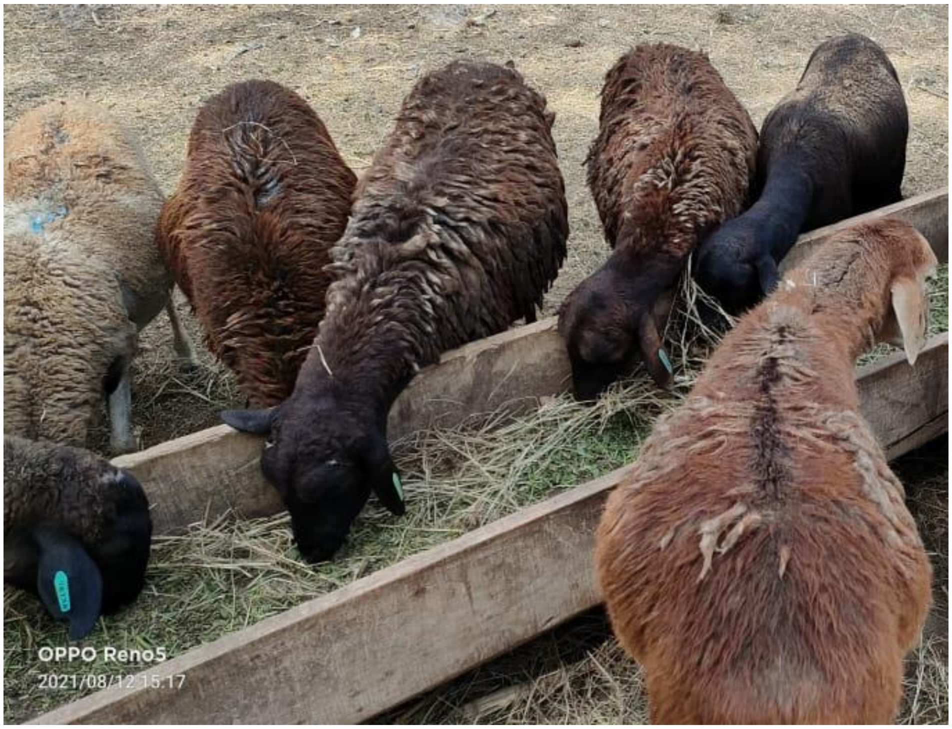

Figure S13: Red Maasai
Photo by George Wanjala

East African fat tail

This is a fat-tailed sheep that serves as dual purpose breed, producing meat and sometimes milk. The breed is named so due to its elongated and fat tail, and it is believed to have originated in the East African region. The tail is believed to serve as a reservoir for adipose tissue, hence its ability to adapt to arid and semi-arid climatic regions. Both males and females are polled, although only a few rams can have horns. Ewes exhibit early reproductive maturity at 18 months at first lambing, and ewes can lamb twice in a year.

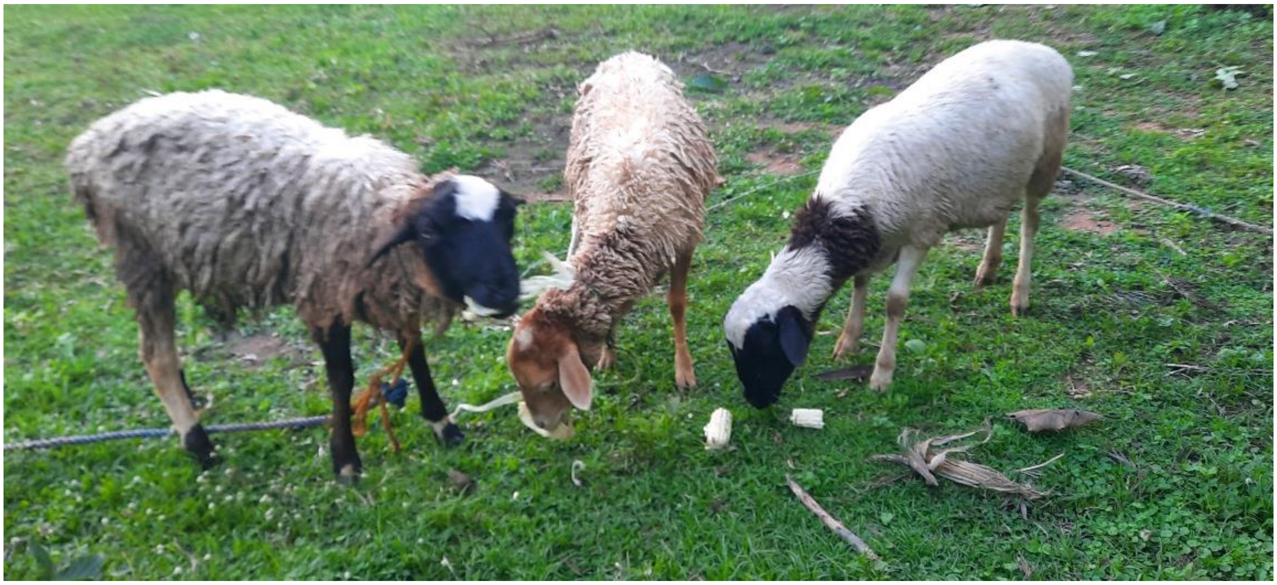

Figure S14: East African fat tail
Photo by George Wanjala

Hungarian breeds

Hungarian Merino

The breed is well adapted to the seasonal changes experienced in Hungary. It was developed by crossing many Merino breeds, mainly to produce wool and milk. However, due to high demand for meat, breeding took the direction of increasing meat productivity and reproduction efficiency. Hungarian Merino has pure white wool with a mutton body conformation. Morphologically, the breed is moderate in body length, has a hairy stomach and legs, and the head protrudes above the top of the ears. Hungarian Merino has a high prolificacy, ranging between 1.3 and 1.5 lambs per lambing. On average, ewes weigh 54 to58 kg, while rams weigh up to 100 kg.

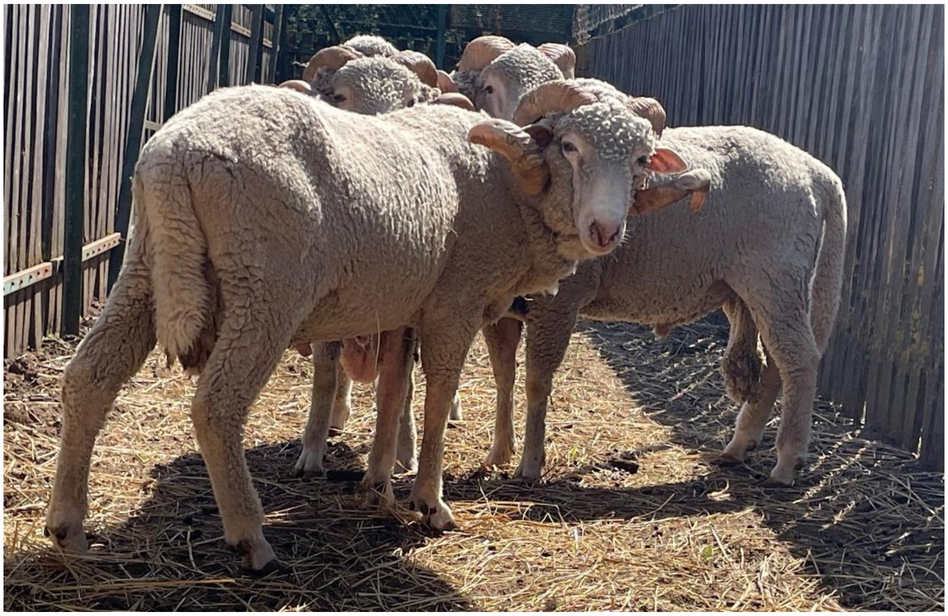

Figure S15: Hungarian Merino
Photo by Zoltán Bagi

Hortobágy Racka

The Racka sheep of Hortobágy is noted for its unusual horns, which are seen on both males and females and have an amazing spiral, corkscrew-like shape. The horns of ewes rise from the head in a V-shaped pattern, but the horns of rams have a wider angle. Rams have an average horn length of around 50 cm, compared to ewes' average horn length of about 30 cm. The breed is distinguished by its small frame with a coat made of tightly coiled, coarse fleece. Black and white are two unique color variants that are separately bred. The breed's head and legs are covered with a thick layer of shiny, tiny, brown, or black hair. Rams normally weigh between 55 and 75 kg, whereas the average weight of ewes is between 35 and 45 kg.

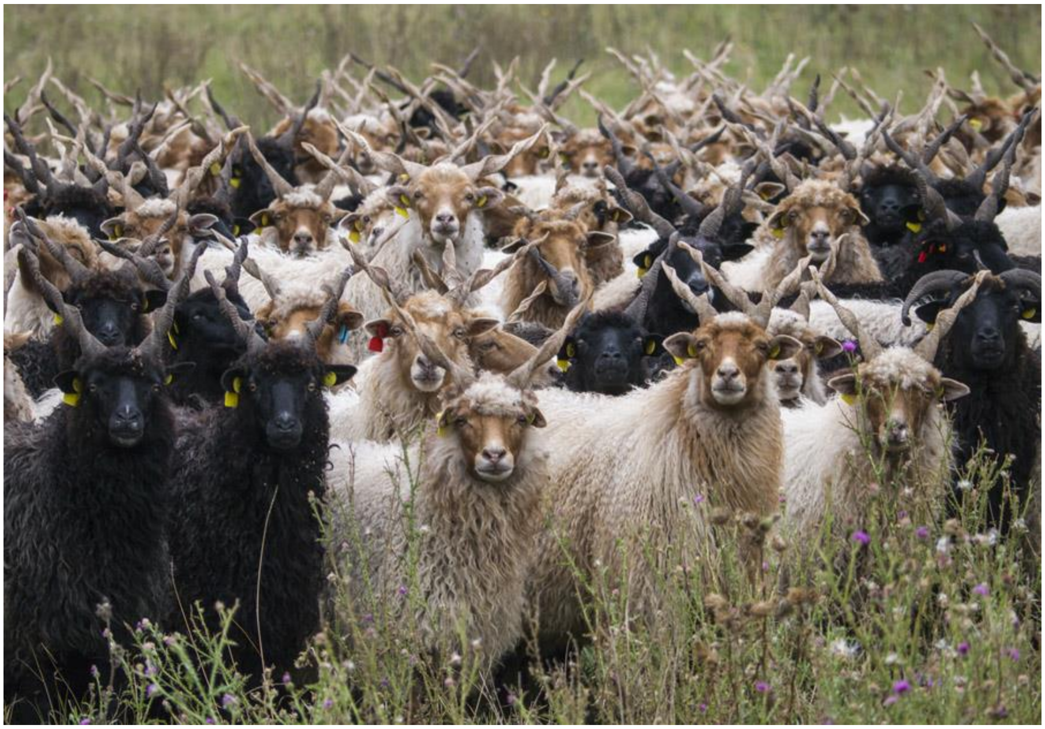

Figure S16: Hortobágy Racka
Photo by Gábor Takács

Hungarian Tsigai

The Tsigai sheep breed, originating from the Balkan Peninsula, was introduced to the Hungarian realm during the late 18th century. This breed is medium, with the facial region and lower extremities exhibiting pigmentation ranging from black to dark brown to brown. The fleece has a mostly white hue, interspersed with threads of various colors. Some ewes are polled, while others are horned with curved horns in a crescent shape. Similarly, rams also exhibit a combination of horned and non-horned individuals. Horned males possess open spiral horns that are characterized by one and a half curls (Fig 12). On average, ewes weigh between 50 and 60 kg, while rams weigh between 75 and 90 kg.

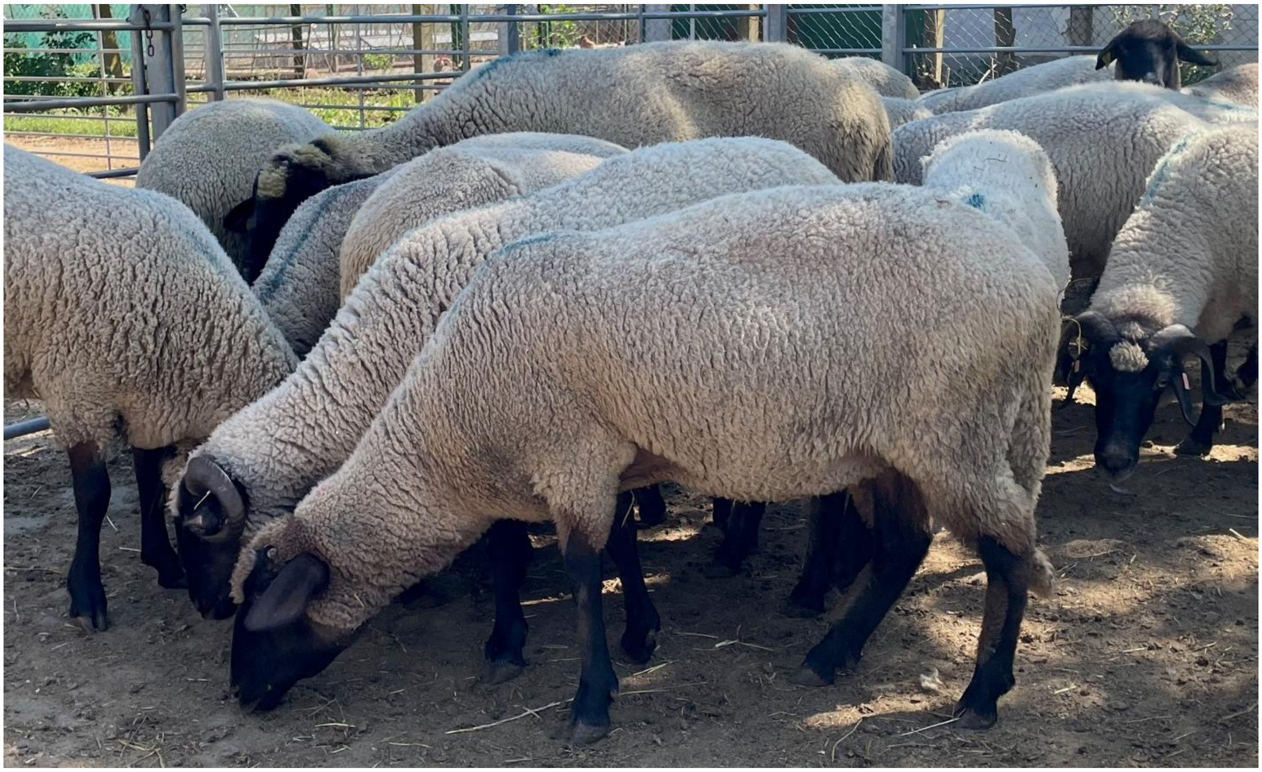

Figure S17: Hungarian Tsigai sheep breed

Photo by Zoltán Bagi

Bábolna Tetra

The Hungarian Bábolna Tetra has been selectively bred from the Hungarian Merino, the Romanov, and the Finnish Landrace breeds. The individuals possess an admirable physical appearance characterized by a well-developed physique and robust skeletal framework. Additionally, they exhibit a lively disposition and resilient overall health. Both genders lack horns. The breed can be bred throughout all breeding seasons of the year and has a potential of producing 1.7 lambs per lambing season. The weight of ewes falls within the range of 50 to 55 kg, whereas males weigh between 65 and 75 kg.

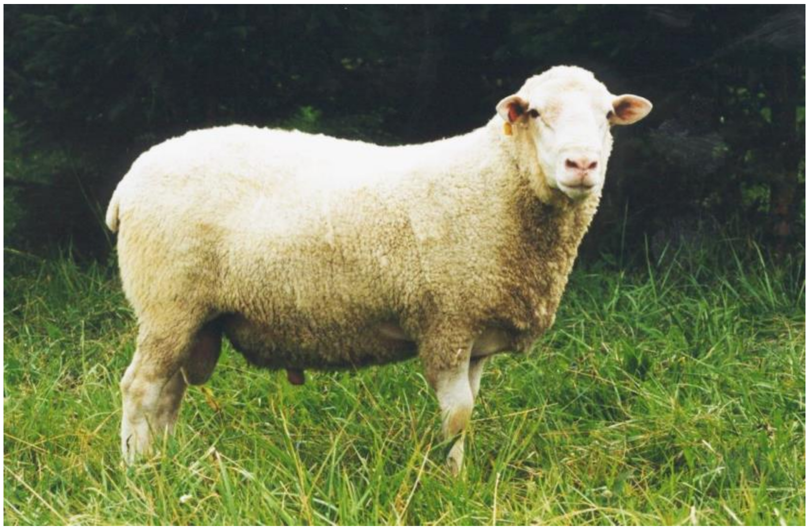

Figure S18: Bábolna Tetra

Ile de France

Ile de France breed has a white coat coloration and possesses muscular body conformation. It shares genetic ancestry with the Merinos. These individuals exhibit favorable maternal traits and have a prolificacy of 1.4 to 1.8. The tendency of this breed to mate outside the breeding season is advantageous, since it allows for breeding throughout the year, a characteristic that distinguishes them from several other mutton breeds. The weight of mature ewes often falls within the range of 70 to 90 kg, whereas males weigh between 100 and 140 kg.

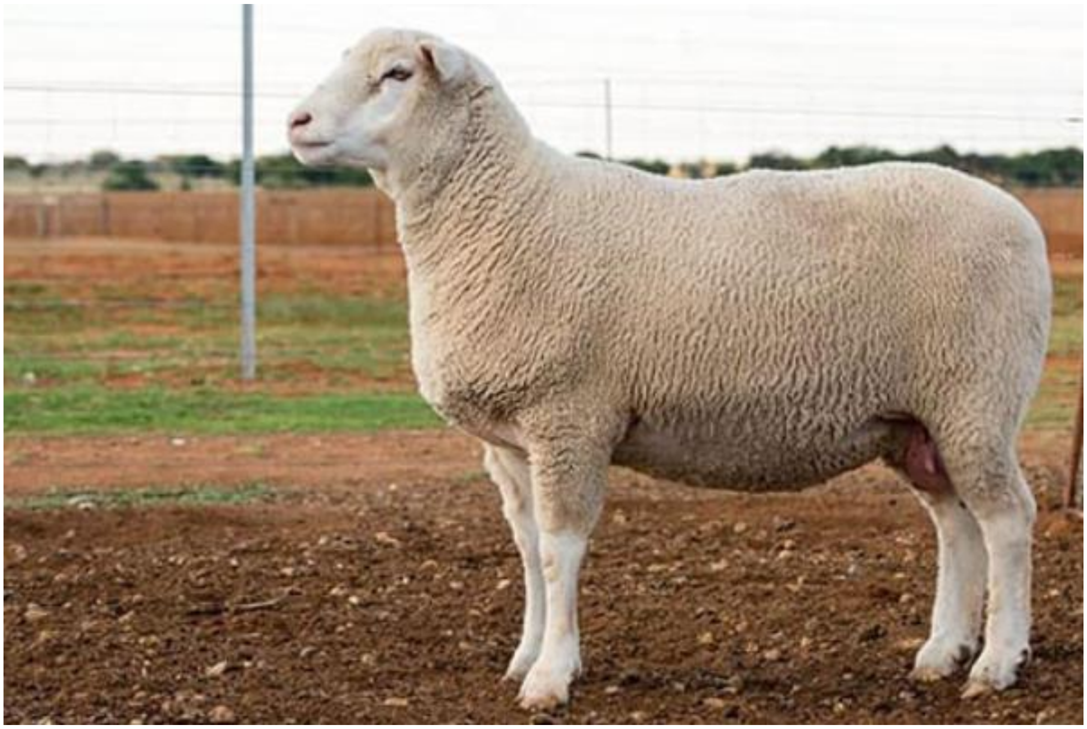

Figure S19: Ile de France

Photo from https://szendroigazdasag.hu/ile-de-france-barany/index.html.

Suffolk

This is a medium-sized breed with a robust body structure and a rapid growth rate. It is primarily bred for the production of mutton and has good grazing behavior. The head and legs have a dense array of shiny, ebony-colored hair. The breed has good maternal traits, good milk production capacity, and a high prolificacy of 1.6 to 1.8 lambs per breeding season. The ability to breed outside the breeding season enables the implementation of an accelerated lambing program. Suffolk has a high level of adaptability to being intensively produced. The breed has favorable meat conformation and body structure. The mature weights of ewes may range from 75 to 90 kg, while males' weight range between 90 and 130 kg.

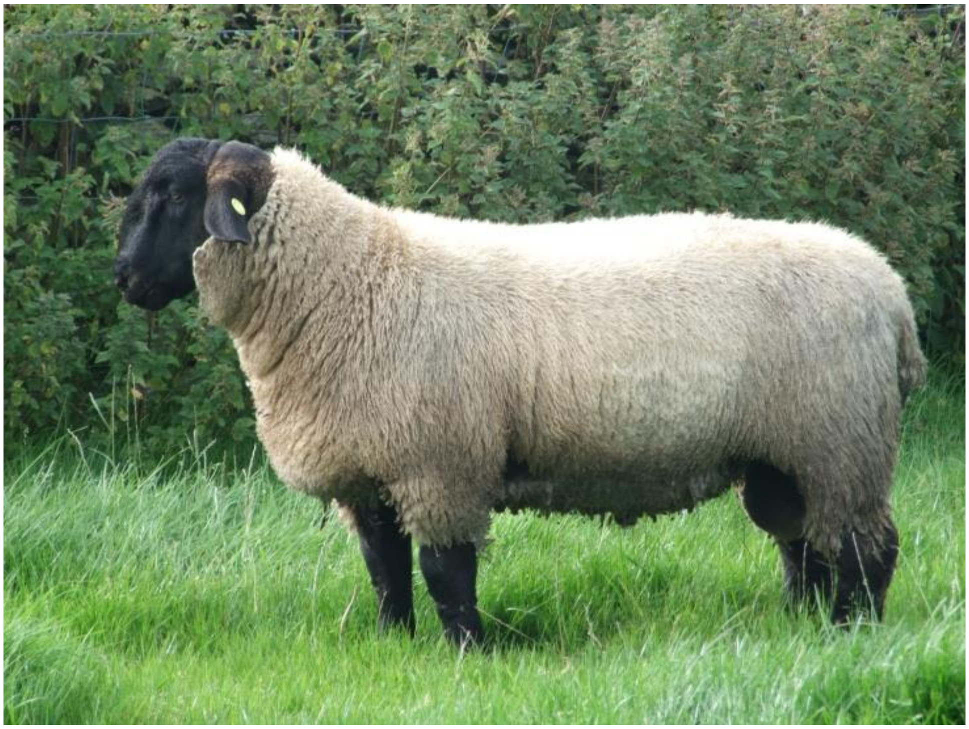

Figure S20: Suffolk
Photo from https://mjksz.com/tenyesztes/fajtak/suffolk.

Bosnian breeds

Dubska Pramenka

Bosnian Dubska Pramenka sheep is a native breed known for milk, meat, and wool production. It was developed in the mid-20th century by crossbreeding French Merinos d'Arles and German Merino-Landschaf breeds. The Dubska Pramenka sheep breed is medium in size with rams weighing 120 kg and ewes 80 kg. Morphologically, the breed has a robust and muscular body. The head may be black or white, whereas the face is mostly black. As opposed to Ewes, rams have horns, and the body is covered with coarse wool. The breed is known for its adaptation to

local environmental conditions, its milk production traits are commendable, while the meat is considered outstanding.

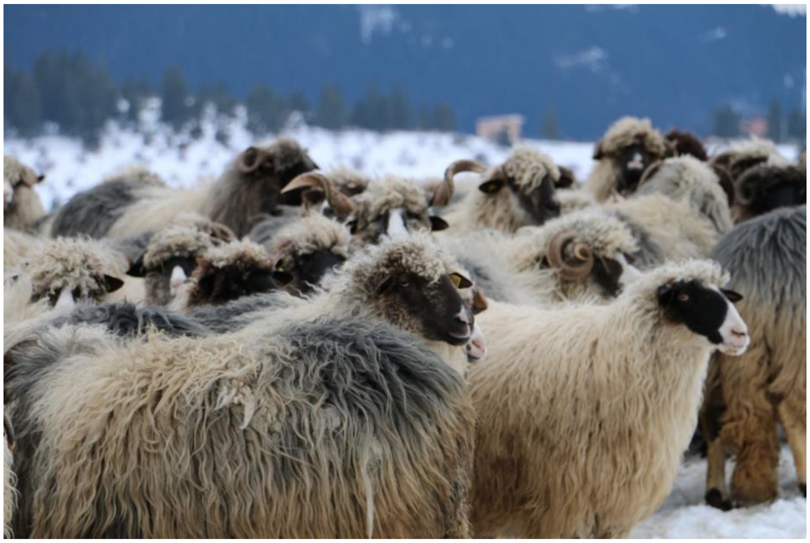

Figure S21: Dubska Pramenka
Photo by Husein Ohran

Hercegovačka Pramenka

Although the breed has been produced in Herzegovina for many years, it was first recognized in 1998. The breed is known for its small frame, with mature males reaching up to 100kg and females weighing up to 60kg. Furthermore, the breed has a muscular body structure with a black head, as are both sides of the face. In contrast to horned rams, the majority of ewes are polled. The Hercegovačka Pramenka breed possesses excellent reproductive characteristics, with ewes reaching sexual maturity at 18 months and a prolificacy of 1.5 lambs per ewe. The Hercegovačka Pramenka sheep is a dual-purpose breed that produces both milk and meat. The milk generated by this breed is used to make cheese.

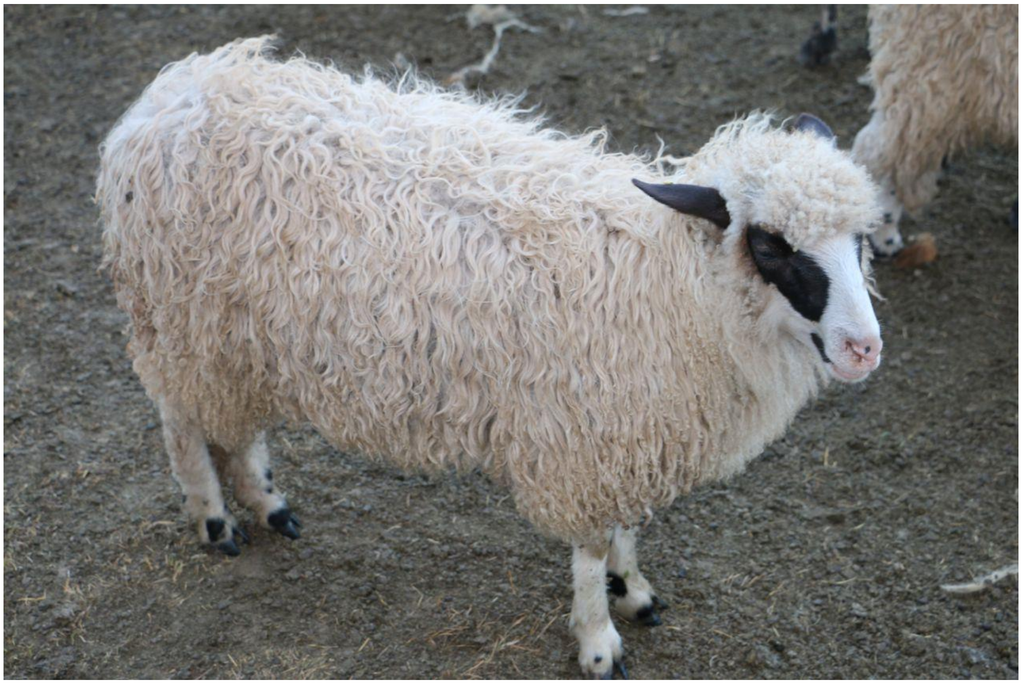

Figure S22: Hercegovačka Pramenka
Photo by Husein Ohran

Kupreška Pramenka sheep

The Kupreška Pramenka sheep is an indigenous breed originating from the region of Bosnia and Herzegovina inhabiting Kupres plateau where the breed is believed to have been developed. The breed received formal recognition in the year 1994. This breed is small in size, males weigh approximately 120 kgs while females weigh about 80 kgs. The head is usually black in color with a varying degree of coloration. Rams of this breed are more often horned while ewes are polled. Ewes typically lamb for the first time at the age of 18 months and have an average lambing rate of 1.8 lambs per ewe. The Pramenka sheep breed is classified as a dual-purpose breed producing milk and meat. The milk produced by this breed is of high quality making it good for cheese production.

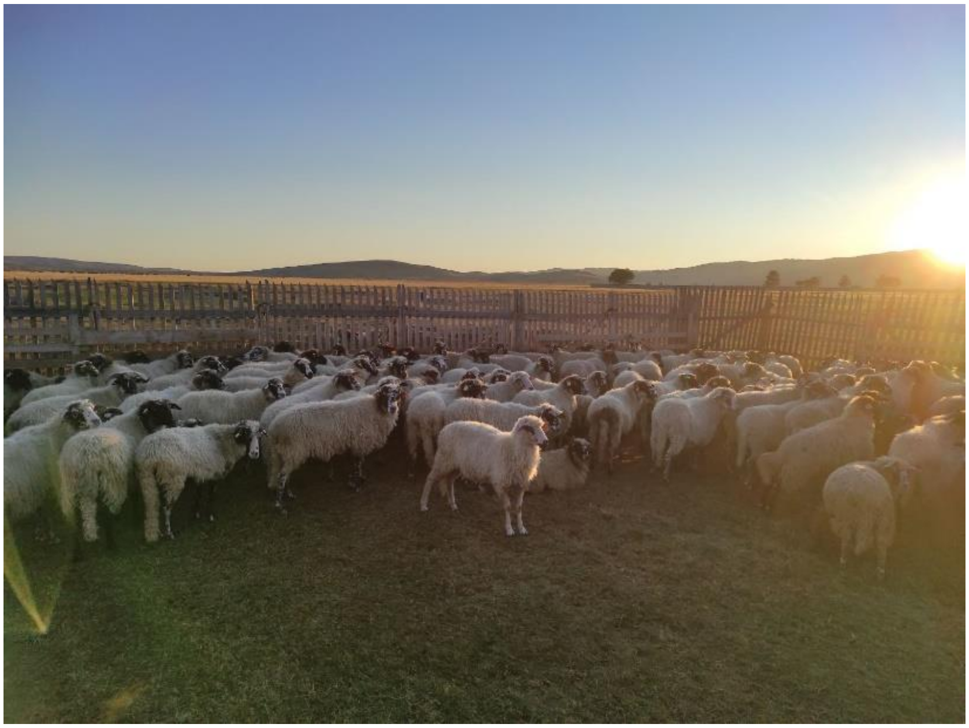

Figure S23: Kupreška Pramenka
Photo by Husein Ohran

Serbian breeds

Lipe sheep/Lipe Pramenka

The Lipe type of Pramenka sheep, is an indigenous breed originating from Serbia and it is potentially endangered. Lipe Pramenka sheep belongs to the category of local type of breeds. It is also called Gara, Garulja by the local people, and represents a triple purpose, late maturing sheep which is today raised under semi-intensive management conditions in the traditional habitat. The breed exhibits distinct traits like a white to grayish white wool coat, a black head, and legs. Ewes are usually polled while horns in rams are prominent and robust, yellowish to dark colored, triangularly shaped, and spirally twisted. The fleece has an open structure characterized by lengthy, flexible locks and coarse fibers. Newborn lambs almost always have black and white coloration. As a Pramenka, it belongs to the long-tailed sheep group Ewes generally weigh around 55 kg, whereas rams are slightly heavier: over 65 kg. Lambs have a birth weight ranging from 3.75 to 5.0 kg. During a lactation period of 120 days, ewes exhibit an average milk production of around 140 liters. The wool production of ewes is about 1.5 kg, whereas rams produce 2.0 kg of wool. The Lipe sheep is described as a local type of the autochthonous Pramenka sheep breed, with robust exterior and good milk properties for cheese production. The breed is reputable for its adaptation to the topographically challenging terrain of Serbia's wetland areas of Pomoravlje.

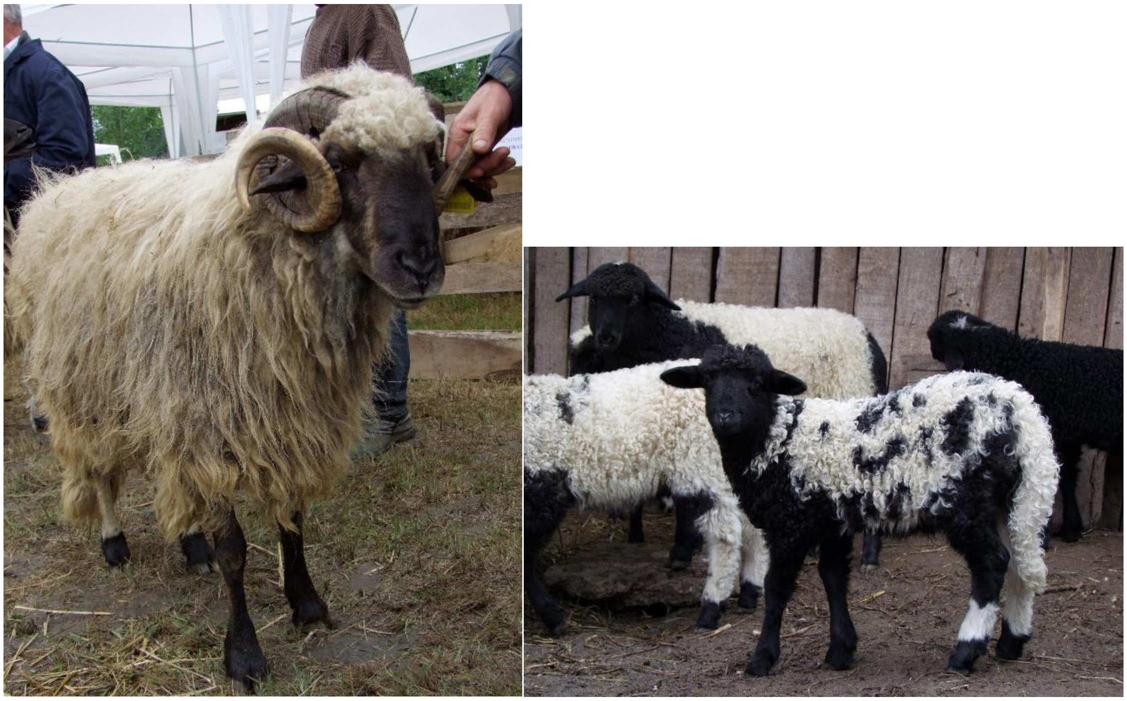

Figure S24: Lipe sheep/Lipe Pramenka
Photo by Zsolt Becskei

Sjenica sheep/ Sjenica Pramenka

Sjenica sheep represents is a transboundary type of Pramenka breed and is a triple product breed, belonging to the long-tailed sheep group. Horns in rams are yellowish to dark, large, triangularly shaped, and spirally twisted, while ewes are usually polled. The profile line is slightly convex. The fleece has white coloration. The head has specific coloration with black hair in the region of nose and mouth, the ears are also black. The black hair around the eyes resembles sunglasses. Black spots are present on the legs. The fleece has a half open structure characterized by flexible locks and coarse fibers. The average weight of mature ewes is 60 kg, whereas rams weigh over 70 kg. The yearly wool production is around 2 kg. The breed is well known for its adaptation to harsh climatic conditions of regions of Sjenica-Pester plateau, where the winter temperatures reach – 40°C and below. The breed is well known as suitable for an extensive breeding system. The famous Sjenica cheese, which has a label of geographical origin, is made of milk from Sjenica sheep.

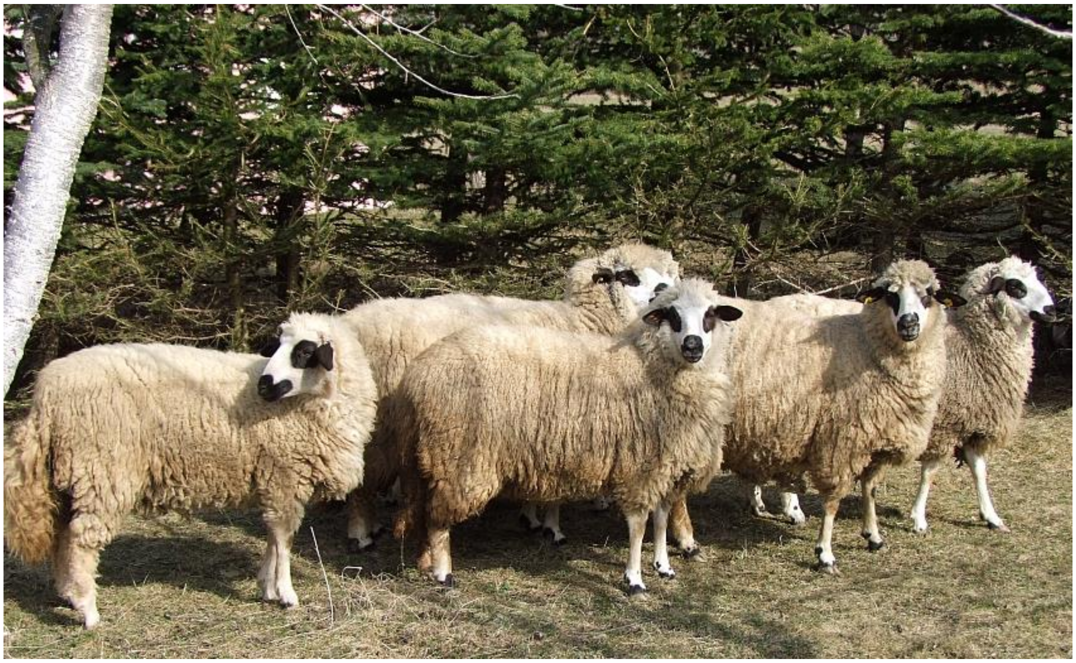

Figure S25: Sjenica sheep/ Sjenica Pramenka
Photo by Zsolt Becskei

South African breeds whose samples were collected from Hungary.

Dorper

This breed was created in South Africa by crossing Dorset Horn and Blackhead Persian sheep. Dorper sheep have black heads and upper necks and white bodies and legs. As hair sheep, Dorper sheep spontaneously lose their fur, removing the need for shearing. They breed year-round including outside breeding seasons and have a prolificacy rate of 160%-180%, making them appropriate for accelerated lambing programs. Dorper sheep are docile and have good maternal traits. They endure drought and good grazing habits. Mature rams weigh 90-110 kg, whereas mature ewes weigh 75-80 kg.

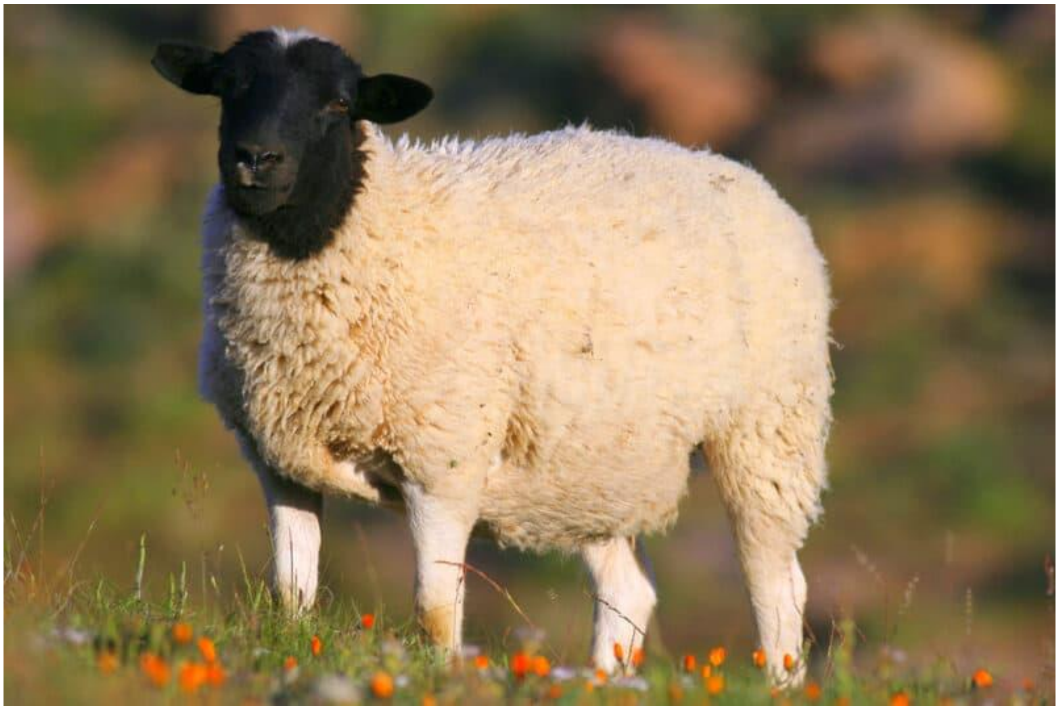

Figure S26: Dorper
Photo from https://mjksz.com/tenyesztes/fajtak/dorper.

White Dorper

This breed was developed in South Africa by the intentional crossbreeding of the Dorper and Afrikaner sheep breeds. The coloration of this breed is white, with the exception of pigmentation present in the claws, under tail region, and udder teats. White dorper is classified as a hair sheep; it mostly sheds its fur and does not need shearing. The breed possesses the ability of breeding year-round, making them well-suited for accelerated lambing programs. Additionally, they have a high level of prolificacy, ranging from 160 to 180%. The breed has a docile and composed disposition and good maternal traits. Their good grazing behavior enables them to adapt to arid and semi-arid climatic conditions. Mature rams weigh 90-110 kg, whereas mature ewes weigh 75-80 kg.

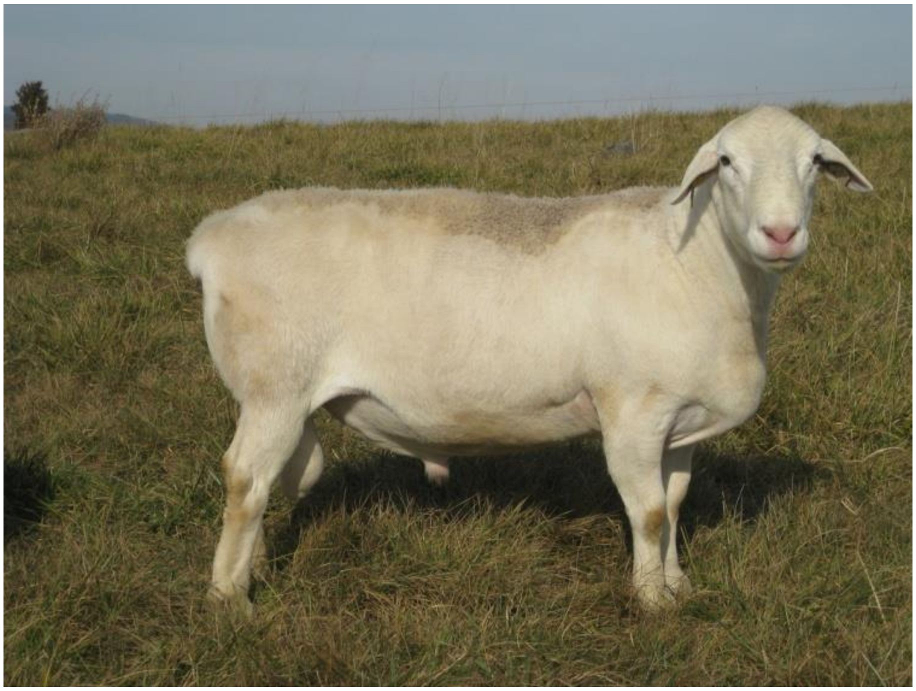

Figure S27: White Dorper
Photo from https://mjksz.com/tenyesztes/fajtak/white-dorper.
