# Supplementary material for: Genetic diversity and adaptability of native sheep breeds from different climatic zones
Source: Sci Rep. 2025 Apr 23;15:14143. doi: 10.1038/s41598-025-97931-2 (PMC12019589; doi:10.1038/s41598-025-97931-2)
Supplement: Supplementary file 5 — Supplementary Material 5 [file 41598_2025_97931_MOESM5_ESM.docx]

**Supplementary information**

Supplementary doc

Format: docx.

Title: Breed description with Figures S1 to S22 showing photos of respectively, Heath Sheep, Podhale Zackel, White-headed Turcana, Rusty Tsigai, Boutisiko, Chios, Lithuanian coarse wool sheep, Red Maasai, East African fat Tail, Hungarian Merino, Hortobágy Racka, Hungarian Tsigai,

Bábolna Tetra, Ile de France, Suffolk, Dubska Pramenka, Hercegovačka Pramenka , Kupreška Pramenka , Lipe Pramenka, Sjenica Pramenka, Dorper and White Dorper

Supplementary File 1 Table S1

Title: Sample description and standard genetic diversity indices (±standard deviation) for all sheep breeds studied.

Supplementary File 1 Table S2

Title: Analysis of molecular variance of all sheep breeds studied.

Supplementary File 1 Table S3

Title: Genes under differencial selection between East Africa and North Africa sheep breeds as identified by Rbs method

Supplementary File 1 Table S4

Title: Genes under differencial selection between East Africa and North Africa sheep breeds as identified by XP-EHH method

Supplementary File 1 Table S5

Title: Genes under differencial selection between East Africa and European sheep breeds as identified by Rsb method.

Supplementary File 1 Table S6

Title: Genes under differencial selection between East Africa and European sheep breeds as identified by XP-EHH method.

Supplementary File 1 Table S7

Title: Genes under differencial selection between North Africa and European sheep breeds as identified by Rsb method.

Supplementary File 1 Table S8

Title: Genes under differencial selection between North Africa and European sheep breeds as identified by XP-EHH method

Supplementary File 2 Figure S 1

Title: Member coefficient for each sheep breed samples from K2 to K35.

(a) Cross validation error graph-lowest point oc the curve is marked “*”; (b) Circular plot showing admixture leves of each breed

Supplementary File 3 Figure S2

Title: Gene interaction network and enrichment terms for genes identified in East Africa vs North Africa sheep breeds.

(a) Gene-gene interaction network for genes identified in East Africa vs North Africa native sheep breeds; (b) A heatmap of Gene-ontology enrichment for genes identified in East Africa vs North Africa sheep breeds

Supplementary File 3 Figure S3

Title: Gene interaction network and enrichment terms for genes identified in East Africa vs European sheep breeds.

(a) Gene-gene interaction network for genes identified in East Africa vs European sheep breeds; (b) A heatmap of Gene-ontology enrichment for genes identified in East Africa vs European sheep breeds

Supplementary File 3 Figure S4

Title: Gene interaction network and enrichment terms for genes identified in North Africa vs European sheep breeds.

(a) Gene-gene interaction network for genes identified in North Africa vs European sheep breeds; (b) A heatmap of Gene-ontology enrichment for genes identified in North Africa vs European sheep breeds

Supplementary File 3 Figures S5

Title: Gene interaction network and enrichment terms for common genes identified in all population pairs.

(a) Gene-gene interaction network for common genes identified in all population pairs; (b) A heatmap of Gene-ontology enrichment for common genes identified in all population pairs.
